# Role of Pleiotropy in the Evolution of a Cryptic Developmental Variation in *Caenorhabditis elegans*

**DOI:** 10.1371/journal.pbio.1001230

**Published:** 2012-01-03

**Authors:** Fabien Duveau, Marie-Anne Félix

**Affiliations:** Institut Jacques Monod, Paris, France; Duke University, United States of America

## Abstract

Robust biological systems are expected to accumulate cryptic genetic variation that does not affect the system output in standard conditions yet may play an evolutionary role once phenotypically expressed under a strong perturbation. Genetic variation that is cryptic relative to a robust trait may accumulate neutrally as it does not change the phenotype, yet it could also evolve under selection if it affects traits related to fitness in addition to its cryptic effect. Cryptic variation affecting the vulval intercellular signaling network was previously uncovered among wild isolates of *Caenorhabditis elegans*. Using a quantitative genetic approach, we identify a non-synonymous polymorphism of the previously uncharacterized *nath-10* gene that affects the vulval phenotype when the system is sensitized with different mutations, but not in wild-type strains. *nath-10* is an essential protein acetyltransferase gene and the homolog of human *NAT10*. The *nath-10* polymorphism also presents non-cryptic effects on life history traits. The *nath-10* allele carried by the N2 reference strain leads to a subtle increase in the egg laying rate and in the total number of sperm, a trait affecting the trade-off between fertility and minimal generation time in hermaphrodite individuals. We show that this allele appeared during early laboratory culture of N2, which allowed us to test whether it may have evolved under selection in this novel environment. The derived allele indeed strongly outcompetes the ancestral allele in laboratory conditions. In conclusion, we identified the molecular nature of a cryptic genetic variation and characterized its evolutionary history. These results show that cryptic genetic variation does not necessarily accumulate neutrally at the whole-organism level, but may evolve through selection for pleiotropic effects that alter fitness. In addition, cultivation in the laboratory has led to adaptive evolution of the reference strain N2 to the laboratory environment, which may modify other phenotypes of interest.

## Abstract 2

Using vulval phenotypes in *Caenorhabditis elegans*, the authors show that cryptic genetic variation can evolve through selection for pleiotropic effects that alter fitness, and identify a cryptic variant that has conferred enhanced fitness on domesticated worms under laboratory conditions.

## Author Summary

Robustness is a property of biological systems that ensures the production of reproducible phenotypes in spite of underlying environmental, stochastic, and genetic variability. A consequence of robustness is that potentially functional genetic variation is free to accumulate in natural populations because it is buffered at the phenotypic level. Even if this so-called “cryptic” genetic variation has no obvious effects under standard conditions, it may become phenotypically expressed upon major genetic or environmental perturbations. Here we used the model organism *Caenorhabditis elegans* to identify genetic variations involved in the cryptic evolution of vulval cell fate induction between wild strains. We found that a mutation in the essential *nath-10* gene not only contributes to cryptic genetic variation in the vulval system, but also affects key life history traits that are expected to be under a strong selective pressure (brood size, age at sexual maturity, sperm number and rate of progeny production). Indeed, an allele of *nath-10* that emerged during the laboratory domestication of *C. elegans* about 50 years ago confers a strong competitive advantage over the ancestral allele under laboratory conditions. A genetic variation that is cryptic for a robust trait can therefore affect more sensitive phenotypes and thus evolve under selection.

## Introduction

Many developmental systems produce outputs that are insensitive to a wide range of environmental or genetic perturbations. In these robust systems, buffering may allow for the accumulation of “cryptic” genetic variation affecting the system without changing its end product [1]–[3]. Upon major environmental or genetic change, genetic variation that was previously cryptic may then become phenotypically expressed and play a role in the evolution of the system [2],[3]. Cryptic variation thus refers to standing genetic variation that is epistatically masked and conditionally neutral: it is not expressed in most conditions, but it can be revealed based on genotype-by-genotype (GxG) interactions with loci involved in the development of the trait or through genotype-by-environment (GxE) interactions, as in the classical experiments by Waddington [4],[5]. Beyond the interest for evolutionary biology, studying this kind of genetic variation is also important to understand the elevated incidence of complex human diseases in modern societies, a phenomenon that could result from the phenotypic expression of cryptic variation in response to the marked change of lifestyle that occurred in the last generations [6].

Two different hypotheses could explain the evolutionary origin of cryptic genetic variation. It may accumulate neutrally because it has little or no effect on traits evolving under selection. Alternatively, a mutation that presents a cryptic effect in a robust system could be under directional selection for its non-cryptic pleiotropic effect on another more sensitive trait. Molecular identification of factors involved in cryptic variation is required to understand the causes and consequences of such variation. Although the empirical study of cryptic genetic variation is limited by the difficulty in detecting it, it has been uncovered in several species after application of a perturbation, either genetic or environmental, to different wild genotypes [7]–[17]. However, in only few cases were the underlying polymorphisms precisely mapped [18] and candidate polymorphisms confirmed [19].

**Figure 1:**
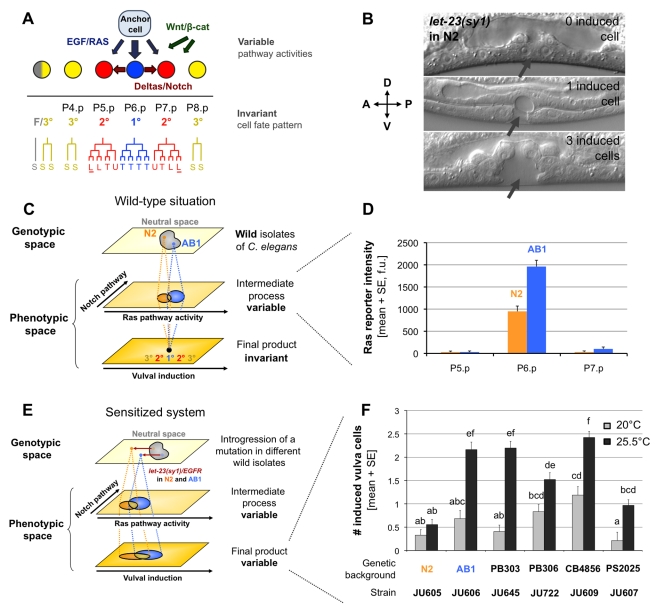
Cryptic variation in *C. elegans* vulval cell fate patterning. (A) Patterning of vulval cell fates in *C. elegans* through an intercellular signaling network. After induction, each cell divides with a characteristic division pattern. Letters indicate the orientation of the last division as follows: T, transverse (left-right); L, longitudinal (anteroposterior); U, undivided. Cells attached to the cuticle are underlined. (S) corresponds to a fusion with the hypodermal syncytium cell hyp7, which is a non-vulval fate. (B) Variable vulval expressivity of the *let-23(sy1)/egfr* sensitizing mutation in the N2 genetic background, observed under Nomarski optics at the L4 stage. The number of induced vulval precursor cells is inferred from their progeny number and morphology in the L4 stage. The expressivity of the mutation differs among genetically identical individuals. The gray arrows point to the expected position of the vulva. (C) Schematic representation of cryptic variation. Genetic variation between wild genotypes leads to variation in intermediate developmental processes such as signaling pathway activities, while the final system output (here vulval cell fates) remains invariant. (D) For instance, the level of expression of the Ras pathway reporter *egl-17::cfp* in P6.p during vulval induction (L3 stage) is about twice lower in the N2 reference strain compared to the AB1 wild isolate [10], yet for both strains the resulting number of induced vulval cells is 3. (E) Cryptic variation is uncovered by sensitizing the system with a mutation. The *let-23(sy1)/egfr* mutation shifts the system outside the buffered region of genotypic space (neutral space). The initially silent difference between N2 and AB1 is now phenotypically expressed at the level of the final product. (F) Expressivity of vulval defects of *let-23(sy1)/egfr* as a function of wild genetic background and temperature. In particular, the N2 genetic background leads to a lower vulval induction index compared to the AB1 background when the system is sensitized, which might be explained by the difference of Ras pathway activity observed in (C). A two-way Scheirer-Ray-Hare extension of the Kruskal-Wallis test detected significant effects of *strain* and *temperature* on the number of induced vulval cells (*strain*: *F* = 15.91, *p* = 0.0005; *temperature*: *F* = 63.69, *p*<0.0001). The genotype-by-environment interaction was not significant in this overall analysis (*strain*×*temperature*: *F* = 1.21, *p* = 0.3386). All pairwise comparisons were performed using Mann-Whitney-Wilcoxon rank sum tests with Holm-Bonferroni method to correct for multiple comparisons. Two bars are significantly different (*p*<0.05) if they are not labeled with a same letter. Error bars indicate the standard error of the mean (SE) over individuals (*n* = 30–72).

The objective of the present study was to characterize the molecular basis and the evolutionary origin of the cryptic variation previously uncovered in the vulval signaling network of the nematode *Caenorhabditis elegans* [10]. The vulva is the egg laying and copulatory organ of *C. elegans* hermaphrodites. It is formed during larval stages from a row of six competent cells in the ventral epidermis (P3.p to P8.p). Only three of these cells (P5.p to P7.p) adopt vulval fates in wild-type animals. P6.p adopts an inner 1° vulval fate, while P5.p and P7.p adopt a lateral 2° vulval fate. After fate specification, each precursor cell follows a specific division pattern during the L3 and L4 stages (Figure 1A). Wild-type vulval cell fate patterning (3°3°2°1°2°3°) relies on the spatio-temporal regulation of a signaling network including the EGF/Ras, Delta/Notch, and Wnt/βcat pathways (Figure 1A) [20]. During the L2 stage, the uterine anchor cell, which is located close to P6.p, starts to emit an inductive LIN-3/EGF signal that can act as a morphogen: the high dose received by P6.p leads to the 1° fate, whereas lower doses received by P5.p and P7.p contribute to their adoption of the 2° fate [21]. Ras/MAP kinase pathway activation in P6.p also promotes the 2° fate in P5.p and P7.p through a lateral Delta/Notch pathway [20]. P3.p, P4.p, and P8.p normally adopt a non-vulval fate, but are able to adopt a vulval fate if P5.p, P6.p, or P7.p are missing. The Wnt/βcat pathway acts at several steps in the process, participating in cell competence, induction, and lineage polarity [22].

The vulval cell fate patterning system displays robustness to both environmental and genetic perturbations, which likely arises from properties of the signaling network creating a non-linear relationship between the activity of individual pathways and the resulting number of induced vulval cells [23]. The cell fate pattern of P4.p to P8.p is quasi-invariant in the *Caenorhabditis* genus and few variants were detected in *C. elegans* individuals raised in various environments, despite change in signaling pathway activity [24]. In addition, a recent computational model was able to reproduce the wild-type vulval cell fate pattern over a broad range of parameter values [25]. As expected in a system with a robust output, the underlying developmental mechanisms have evolved at the interspecific and intraspecific levels (Figure 1C). Indeed, quantitative variation in a downstream fluorescent reporter of the EGF/Ras pathway was detected among *C. elegans* wild isolates despite no change in the resulting cell fates (Figure 1D) [10]. Furthermore, the type and frequency of vulval fate errors following a variety of environmental, genetic, or experimental perturbations differ among *Caenorhabditis* species and *C. elegans* wild isolates (Figure 1E–F) [10],[24],[26]–[28]. Due to the broad knowledge of the developmental network, the small number of cells involved and the ease of genetic studies in *C. elegans*, vulval cell fate patterning is an ideal model to investigate the evolutionary origin and consequences of cryptic variation.

One of the genetic perturbations used to reveal cryptic variation among *C. elegans* wild isolates was the *let-23(sy1)/egfr* allele (Figure 1B) [10]. This EMS-induced mutation alters EGF receptor localization in vulval precursor cells and leads to a decrease in the mean number of cells adopting a vulval fate [29],[30], as well as an increase in the among-individual variation due to a shift of the system outside its buffered range. The expressivity of *let-23(sy1)* varies among wild genetic backgrounds of *C. elegans*, revealing cryptic variation among them [10]. Especially, *let-23(sy1)* expressivity, as quantified by the mean number of cells adopting a vulval fate (vulval index), is much less pronounced in the AB1 wild background than in the N2 reference background in which this mutation was isolated (Figure 1F) [10]. In this case, the effect of the cryptic variation is thus revealed through an epistatic interaction between the laboratory allele at the *let-23* locus and the wild genetic background.

Here, we use a quantitative genetic approach to characterize the genetic architecture of variation in *let-23(sy1)* expressivity between these wild genetic backgrounds and identify underlying molecular polymorphisms. We find that a non-synonymous polymorphism in the *nath-10* gene explains a major effect quantitative trait locus (QTL). *nath-10* is the human N-acetyltransferase 10 homolog and its function is largely unknown in *C. elegans*. We show that the *nath-10(N2)* allele likely appeared during early laboratory culture of the N2 strain and that in addition to its cryptic effect in the vulval system, this allele presents non-cryptic effects on life history traits such as progeny number, minimal generation time, or egg-production rate. Some of the effects of the *nath-10* polymorphism are likely mediated through alterations in the timing of the sperm/oocyte switch in hermaphrodites. Finally, competition assays show a clear selective advantage of the N2 allele in laboratory conditions. We thus identify a pleiotropic polymorphism that shows a cryptic effect in the vulval system and a strong selective advantage in the laboratory environment due to its effect on life history traits. Therefore, because of pleiotropic effects, some cryptic genetic variation need not accumulate neutrally during the evolution of robust systems.

## Results

### Temperature Sensitivity of the Intraspecific Variation Uncovered by the *let-23(sy1)*/*egfr* Mutation

As previously observed [10], the expressivity of vulval induction defects of a *let-23(sy1)/egfr* mutation was much higher in the JU605 strain (N2 background) than in the JU606 strain (AB1 background) (Figure 1E, Table S3). We express this phenotype as a vulval index, corresponding to the mean number of Pn.p cells induced to adopt a vulval fate in different individuals of a given strain.

We repeatedly observed that the vulval index of the JU606 isogenic strain could significantly vary among cultures grown at 20°C and scored in parallel (Figure S1). Therefore, we searched for culture conditions in which the vulval phenotype of JU606 would be less variable. We found that the vulval index of JU606 was consistently increased at 25.5°C (but not at 24°C) compared to 20°C, whereas the vulval index of JU605 was not affected (Figure 1F, Figure S1). We therefore used this culture temperature to map the genetic variation involved in the phenotypic difference.

### Genetic Architecture of the Cryptic Variation

**Figure 2:**
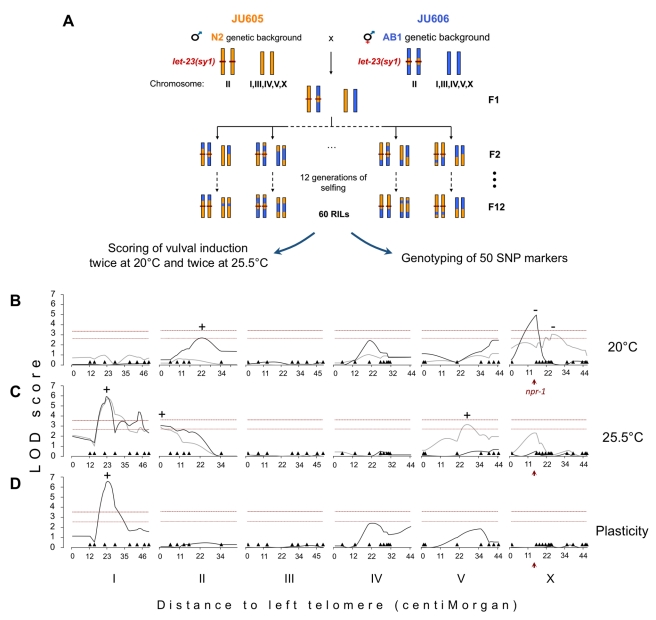
Genetic architecture of the cryptic variation uncovered by *let-23(sy1)/egfr* in the N2 and AB1 backgrounds. (A) Recombinant inbred lines (RILs) construction scheme. Examples of possible strain genotypes are schematized, with the N2 genetic background colored in orange, AB1 in blue, and the *let-23(sy1)* mutation as a red line. (B–D) Logarithm of odds (LOD) plots showing composite interval mapping of vulval index in RILs grown at (B) 20°C or (C) 25.5°C and for (D) the plasticity of vulval index to temperature. The gray and black curves represent two replicates of phenotypic scoring. In the plasticity mapping, a single replicate (dark gray) was used, for which vulval index scoring was performed in parallel at both temperatures. The plasticity measure was obtained by substracting for each RIL the vulval index at 25.5°C to that at 20°C. (B–D) Dark triangles show marker positions along the chromosomes. Horizontal dashed lines indicate the 1% (top) and 5% (bottom) significance thresholds computed by multiple permutations. (+) and (−) represent the direction of QTL effect. (+) means that AB1 alleles at QTL lead to higher trait value than N2 alleles and (−) the reverse. The red arrowhead points to the position of *npr-1* gene.

In order to characterize the genetic architecture underlying the variation in vulval index between JU605 and JU606, we constructed a set of 60 Recombinant Inbred Lines (RILs) from a cross between the two strains. The RILs were genotyped for 50 N2/AB1 SNP markers distributed along the genome (Table S1). The vulval index of all lines was scored twice at both 20°C and 25.5°C (Figure 2A; Table S1). Statistical analyses were performed to detect quantitative trait loci (QTL), which are genomic regions significantly associated with phenotypic variation. Two QTLs were detected at 20°C (Figure 2B), but their effect was not reproducible between the two replicates, probably due to the high vulval index variability at this temperature (Figure S1). By contrast, two QTLs were reproducibly found on chromosomes I and II at 25.5°C (Figure 2C). The QTL on chromosome I was estimated to explain about 27% to 34% of the phenotypic variance, while the effect of the chromosome II QTL was only about 3.6% to 4.6% (Multiple Interval Mapping, unpublished data). Another QTL was observed in one replicate on chromosome V at this temperature, but was not reproducible (Figure 2C). AB1 alleles at these QTLs all led to higher vulval index than N2 alleles (positive effect QTL). Finally, a QTL with strong negative effect was detected in one replicate at 20°C. This negative effect QTL could contribute to the transgressive phenotype of some RILs that presented a more extreme vulval induction index than either parental strain (Table S1). The *npr-1* polymorphism [31] was a candidate to explain this putative QTL because it was previously identified in QTL analyses of various traits [32]–[36]. In support of this hypothesis, strains carrying the N2 allele of *npr-1* in the N2 background presented a higher vulval index at 25.5°C than strains with the AB1 allele *npr-1(g320)* introgressed in the N2 background (Figure S2). This effect seemed sensitive to environmental conditions because it was not always observed in replicate experiments at 20°C or at 25.5°C (unpublished data and Figures 2B–C, S2).

In order to characterize the genetic basis of the phenotypic plasticity to temperature, an additional QTL analysis was performed using as trait value for each RIL the difference in vulval induction index between 25.5°C and 20°C (Figure 2D). The only QTL that was detected in this analysis corresponded to the major effect QTL detected on chromosome I at 25.5°C, which was absent in the 20°C analyses. This plastic effect corresponds to a genotype-by-environment interaction (GxE) since the effect of the QTL allele on vulval induction depends on temperature.

Together, these results showed that the genetic architecture of the phenotypic variation observed between JU605 and JU606 was temperature-sensitive and involved more than one locus. No significant epistatic interactions were detected between single-effect QTLs (multiple interval mapping, unpublished data).

### Cryptic Effect of *nath-10* Polymorphism on Vulval Induction

**Figure 3:**
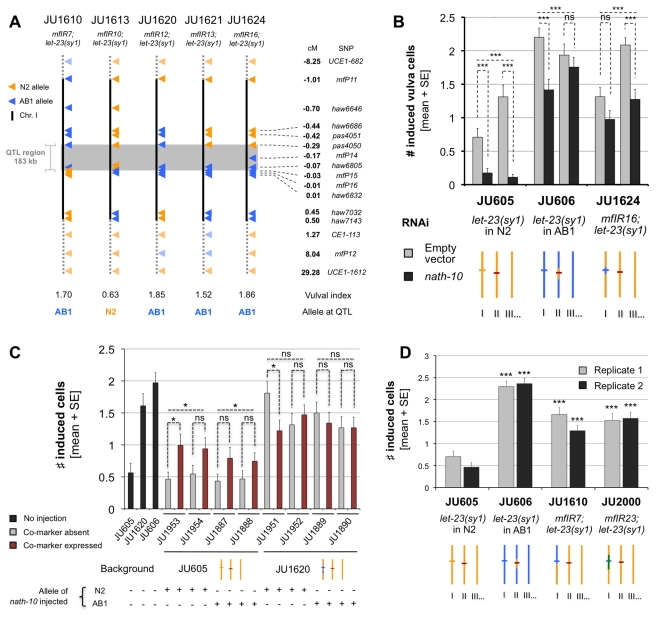
A polymorphism in the *nath-10* gene explains the largest effect QTL. (A) Fine-mapping of the QTL to a 183 kb region (gray rectangle). Chromosome I genotypes of five near-isogenic lines (NILs) at SNP markers (triangles) are represented. Orange triangles, N2 alleles; blue, AB1. Their vulval index at 25.5°C is indicated below. Genetic distances are represented to scale along full black lines, but not outside. The vulval index is significantly lower in JU1613 compared to the other NILs (Mann-Whitney-Wilcoxon rank sum tests, *p*<10^−4^, *n* = 30–42). (B) RNAi against *nath-10*. The significance of pairwise Mann-Whitney-Wilcoxon rank sum tests comparing the *nath-10* RNAi versus control bacteria is represented above each pair of bars (*n* = 51–64). Combined *p* values of replicates using Fisher's method is indicated above. (C) Overexpression of N2 and AB1 *nath-10* alleles. Worms that spontaneously lost the transgene (gray) were compared to sibling worms that retained it (red). Black bars represent non-injected strains. Two replicates were scored per injected strain and allele (*n* = 36–45). Pairwise comparisons using Mann-Whitney-Wilcoxon rank sum tests were only significant in one case. However, combining the results using Fisher's method (top) showed a significant effect of each allele when injected into JU605, but not into JU1620. (D) Introgression of the *nath-10* allele from LSJ1 into N2. The vulval index of JU606, JU1610, and JU2000 was compared to N2 in two replicate experiments using Mann-Whitney-Wilcoxon rank sum tests. (B–D) Vulval phenotypes were scored in animals grown at 25.5°C. ns, non-significant, * 0.01<*p*<0.05, *** *p*<0.001. Strain genotypes are schematized below the graphs, with orange for N2 and blue for AB1 background. Orange and blue horizontal lines on chromosome I represent *nath-10* alleles and the red line on chromosome II *let-23(sy1)*. Only one chromosome copy is shown (the strains are rendered homozygous through selfing).

Since the QTL detected at 25.5°C on chromosome I was involved in a major fraction of the phenotypic variation, we next sought to identify the underlying causative molecular polymorphism(s). Several lines were selected for a recombination event in the chromosome I QTL region after crosses of the RILs with highest vulval index to JU605 (*let-23(sy1)* in N2). After SNP genotyping and scoring of vulval index in these lines (Table S2), the QTL was restricted to a 183 kb region (Figure 3A; Table S3). The alignment of the N2 reference genome (www.wormbase.org) to the genome of the AB1 strain (Illumina sequencing [L. Stein et al., personal communication] followed by Sanger sequencing of non-covered regions) revealed the presence of only three polymorphisms in the QTL region. Two of them lie in intergenic regions (*mfP14* and *haw6786*) and the third one (*haw6805*) affects the coding sequence of a gene with unknown function, *F55A12.8*, which was renamed *nath-10* for its homology with the human *NAT10* gene (*N-acetyltransferase 10*). We performed several experiments that test whether the *nath-10* polymorphism explains the chromosome I QTL effect.

First, RNAi inactivation of *nath-10* in lines carrying either the *nath-10(N2)* or the *nath-10(haw6805)* allele decreased the vulval index (Figure 3B). This suggested that *nath-10* acts as a positive regulator of vulval induction and that *nath-10(N2)* is a hypomorphic allele compared to *nath-10(haw6805)*. In addition, the effect of *nath-10(N2)* on vulval induction was found to be recessive to that of *nath-10(haw6805)* (Figure S3A). The vulval index still differed after *nath-10(RNAi)* between the JU605 (*nath-10(N2)*) and the JU1624 (*nath-10(haw6805)*) strains (Figure 3B), which may be explained by a partial inactivation of *nath-10* by the RNAi treatment or by parental effects; alternatively, another polymorphism in the introgressed AB1 region of JU1624 could affect vulval induction. Heterozygous animals carrying the *nath-10(N2)* allele over the null mutation *nath-10(tm2624)* presented a decreased vulval index compared to JU605 (Figure S3B). However, it is not clear whether this was due to the *tm2624* deletion or to the translocation used to balance this embryonic lethal mutation (Figure S3B).

In order to confirm the role of *nath-10* in vulval induction, we also overexpressed the gene using transgenesis. Overexpression of either *nath-10(haw6805)* or *nath-10(N2)* alleles increased the vulval index in the JU605 strain but not in the JU1620 (*nath-10(haw6805)*) strain (Figure 3C). These results indicate that both alleles are functional, consistent with *nath-10(N2)* being a hypomorph and not a null allele. A possible explanation for the absence of effect of *nath-10* overexpression in the JU1620 strain background may be that the endogenous *nath-10(haw6805)* activity is already saturating. Alternatively, *nath-10* overexpression may have weak effects due to the low concentration of *nath-10* genomic DNA used to avoid the lethality observed at higher concentrations. This may also explain why *nath-10(haw6805)* overexpression does not rescue vulval induction in JU605 to the level observed in JU1620 (Figure 3C).

A third experiment was performed to test whether the two other polymorphisms in the QTL region (Table S2) could be ruled out. The *nath-10* polymorphism was the only variation with N2 found to be shared by the LSJ1 and AB1 strains in the QTL region (LSJ1 is a strain closely related to N2, as described below) [37],[38]. The *nath-10(haw6805)* allele was thus introgressed into JU605 (*let-23(sy1)* in N2) from the LSJ1 genetic background. Irrespective of their genetic background origin, strains homozygous for *nath-10(haw6805)* always presented a higher induction index than strains homozygous for *nath-10(N2)* at 25.5°C (Figures 3D, S5B). Therefore, the QTL effect is very probably due to the *nath-10* polymorphism and not to other polymorphisms in the QTL region.

From the three experiments above, we conclude that *nath-10(haw6805)* is the causative chromosome I polymorphism that explains a large part of the difference in expressivity of the *let-23(sy1)* allele between the N2 and AB1 genetic backgrounds. The increase in vulval index conferred by the *nath-10(haw6805)* allele represented 53%±4% of the total difference observed between the JU605 and JU606 parental strains.

### Effect of *nath-10* Polymorphism on the Expressivity of a *let-60(gf)* Mutation

**Figure 4:**
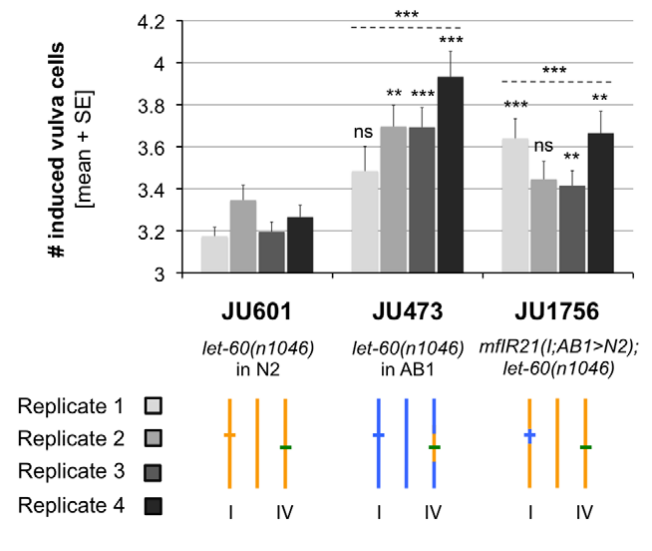
Effect of the QTL on expressivity of *let-60(n1046)/ras*. The bar shades represent four independent replicates. Mann-Whitney-Wilcoxon rank sum tests followed by Holm-Bonferroni correction for multiple testing were used to test for significant differences of strains carrying the AB1 QTL allele (JU473 or JU1756) with the strain carrying the N2 allele (JU601). ns, non-significant; ** *p*<0.01, *** *p*<0.001 (*n* = 21–46). Stars above dotted lines represent the combination of the *p* values obtained for the four replicates using Fisher's method. Genotypes are schematized as in Figure 3, except that green lines indicate *let-60(n1046)*.

The *nath-10* polymorphism does not affect vulval cell fates in a wild-type context (no defect observed in N2 and AB1 individuals grown at 25.5°C, *n* = 100). We wondered whether its effect on *let-23(sy1)* expressivity was specific to the sensitizing mutation. To address this point, a gain-of-function mutation in Ras, *let-60(n1046)/ras*, was crossed in the N2 background with the introgressed segment *mfIR16* bearing the *nath-10(haw6805)* allele from AB1. *let-60(n1046gf)* led to vulval fate hyperinduction (more than 3 induced Pn.p cells), and the vulval index was higher in lines carrying the *nath-10(haw6805)* allele (Figure 4), as was the case for *let-23(sy1)*. Therefore, the effect of the *nath-10* polymorphism can be uncovered with different sensitizing factors and is not specific to the mutation initially used to identify it.

### The *nath-10* Polymorphism Affects the Peptide Sequence of the *C. elegans* Homolog of Human N-Acetyltransferase 10

**Figure 5:**
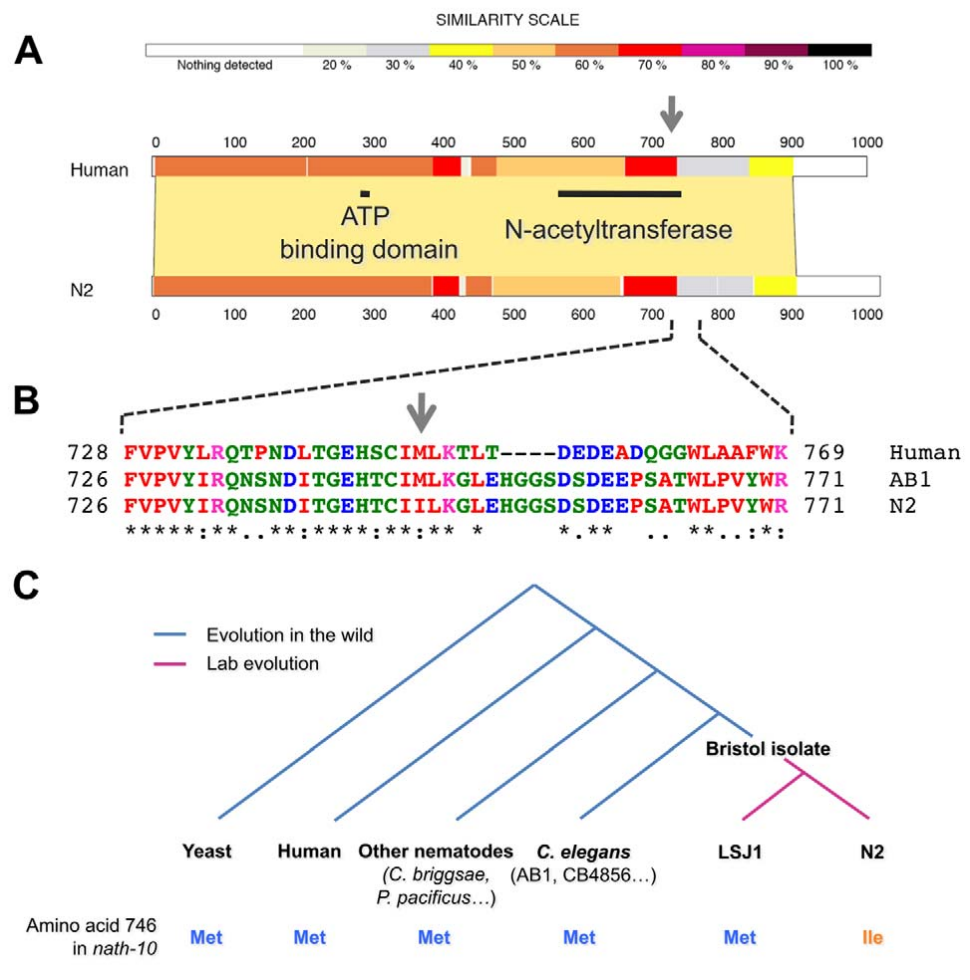
The coding *nath-10* polymorphism evolved during laboratory domestication of the N2 reference strain. (A) Pairwise alignment of human NAT10 and N2 NATH-10 protein sequences. Horizontal dark lines represent the GNAT-related N-acetyltransferase domain (amino-acids 558–753) and a putative ATP binding domain (amino acids 284–291). The gray arrow points to the position of the *haw6805* polymorphism. (B) Close-up on amino-acid alignment in the vicinity of the *haw6805* polymorphism (gray arrow). Colored letters illustrate residue properties. Red, small, hydrophobic, aromatic, not Y; blue, acidic; magenta, basic; green, hydroxyl, amine, amide, basic. (C) Distribution of the *nath-10* polymorphism among different *C. elegans* wild strains and eukaryote species.

*nath-10* encodes a polypeptide of 1,043 amino acids. Pairwise alignment of the NATH-10 protein sequence with its human homolog NAT10 indicates a 57.3% conservation at the amino-acid level (Figure 5A). In human, the NAT10 protein regulates different cellular processes such as cytokinesis, mitotic chromosome decondensation, or telomerase expression and it can acetylate different substrates such as histones and α-tubulin [39]–[42]. In *C. elegans*, *nath-10* was recently identified in a RNAi screen for abnormal expression of sex-specific gonadal markers [43], but its function has not been further investigated. The recessive *nath-10(tm2624)* deletion, which results in a truncated protein of 394 amino-acids, leads to fully penetrant embryonic lethality. The N2 protein presents an isoleucine residue at position 746 instead of the methionine found in the AB1 protein or in the human alignment at the corresponding position (Figure 5B). This non-synonymous polymorphism affects a conserved region, which corresponds to the acetyltransferase domain in the human counterpart (Figure 5A).

A protein with acetyltransferase activity could act at different levels of the vulval signaling network to regulate cell fate induction. For instance, *mys-1* and *hda-1*, which encode a MYST family histone acetyltransferase and a class I histone deacetylase, respectively, can both act as class B SynMuv genes to repress vulval induction [44]–[46]. Single mutations of SynMuv genes do not affect vulval fate patterning, whereas the combination of two mutations belonging to different SynMuv classes result in a Synthetic Multivulva phenotype. Remarkably, several SynMuv B null mutants respond to temperature in a similar way as *nath-10(haw6805)* animals: they present a different phenotype at 24°C compared to 25–26°C [47],[48]. For these reasons, we tested whether the combination of the *nath-10(haw6805)* allele with a SynMuv mutation of either class led to excess vulval induction. The *nath-10(haw6805)*; *lin-15A(n767)* and *nath-10(haw6805)*; *lin-15B(n744)* lines did not present a Multivulva phenotype (Table S4), suggesting that *nath-10* does not act as a SynMuv gene.

### The *nath-10(N2)* Allele Appeared During Laboratory Domestication of the N2 Reference Strain

To study the evolutionary history of the *nath-10* polymorphism, we determined the allelic distribution in a set of *C. elegans* natural isolates and related species (Figure 5C, Table S5). The N2 allele was only found in two other strains, CB4555 and TR389, which were likely derived by contamination from the N2 lab reference strain [49]. The AB1 allele was found in all other *C. elegans* isolates and conserved in other nematode species, fly, human, or yeast. Thus, *nath-10(N2)* is a derived allele relative to the ancestral *nath-10(haw6805)* allele.

The observed polymorphism distribution raised the possibility that the *nath-10(N2)* allele did not appear in the wild. This hypothesis was confirmed by the presence of *nath-10(haw6805)* in the LSJ1 laboratory strain, which is likely derived from the same Bristol wild isolate as N2 [34],[37],[38]. The so-called “N2 (ancestral)” strain (CGC), which is only about six generations away from the earliest frozen stock of N2, presents the *nath-10(N2)* allele (Table S5). The *nath-10(N2)* allele thus most probably arose in a period spanning the initial separation of the N2 and LSJ1 strains in the Dougherty lab at UC Berkeley (between 1957 and 1960) to the first freezing of N2 in Sydney Brenner's laboratory around 1968.

### Non-Cryptic Effects of the *nath-10* Polymorphism on Life History Traits

With the aim of understanding the evolutionary factors underlying the fixation of the *nath-10(N2)* allele in the N2 strain, we enquired whether other traits were affected by the *nath-10* polymorphism in the absence of the *let-23(sy1)* sensitizing mutation. We focused on life history traits that were known to vary among wild isolates and were likely to affect individual fitness, such as the environmental regulation of dauer diapause entry, lifetime fecundity, minimal generation time, or egg laying rate. We observed that the frequency of dauer formation at 27°C [50] was much higher in AB1 than in N2. However, two lines carrying the *nath-10(haw6805)* allele introgressed into the N2 background did not display any phenotypic difference for this trait compared to N2 (Table S6). Therefore, the dauer entry variation between N2 and AB1 is not caused by the *nath-10* polymorphism.

**Figure 6:**
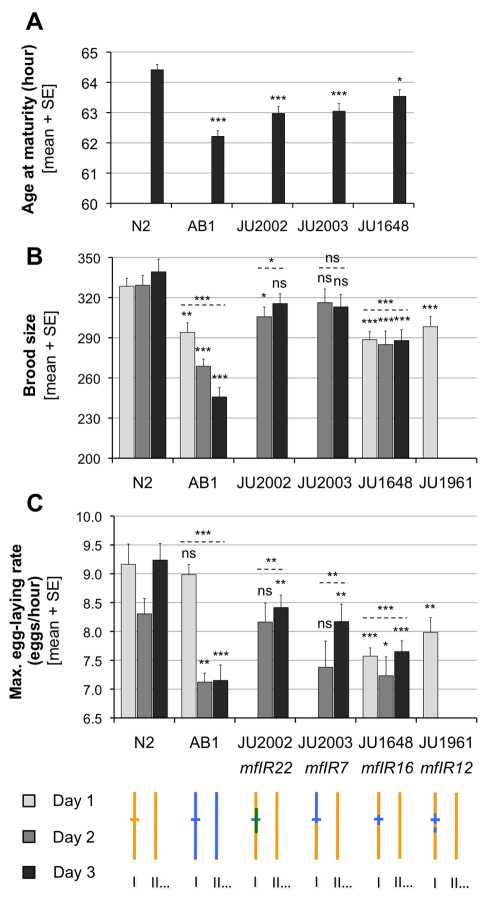
Non-cryptic effect of the *nath-10* polymorphism on life history traits. (A) Minimal generation time at 20°C (*n* = 19–20). (B) Brood size at 20°C (*n* = 17–20 parents). (C) Maximal egg laying rate at 20°C (*n* = 17–20). Each strain carrying *nath-10(haw6805)* (and no sensitizing mutation) was compared to N2 (Mann-Whitney-Wilcoxon rank sum test). Bars of different shades represent replicates, with the same shade in different panels indicating that the same individuals were scored for different traits. The *p* values of replicates were combined using Fisher's method and represented above the dotted lines. ns, non-significant; * *p*<0.05, ** *p*<0.01, *** *p*<0.001. Genotypes are schematized below as in Figure 3.

In contrast to dauer formation, the *nath-10* polymorphism resulted in variation in three key traits related to population growth: age at maturity, brood size, and egg laying speed (Figure 6A–C). These traits were measured in parallel in N2, AB1, and near isogenic lines with the *nath-10(haw6805)* allele introgressed from the AB1 background into the N2 background (JU2003, JU1648, and JU1961) or from the LSJ1 background into the N2 background (JU2002). The introgressions were performed from these two genetic backgrounds to ensure that any phenotypic difference with N2 could be attributed to *nath-10* polymorphism and not to other polymorphisms in the introgressed regions (Table S2). N2 presented a significantly higher age at maturity (Figure 6A), brood size (Figure 6B), and maximal egg laying rate (Figure 6C) compared to AB1. For all three traits, a significant, albeit sometimes lower difference with N2 was also observed in the introgressed *nath-10(haw6805)* lines. These results strongly suggest that in addition to its cryptic role on vulval induction, the *nath-10* polymorphism also affects some life history traits non-cryptically.

**Figure 7:**
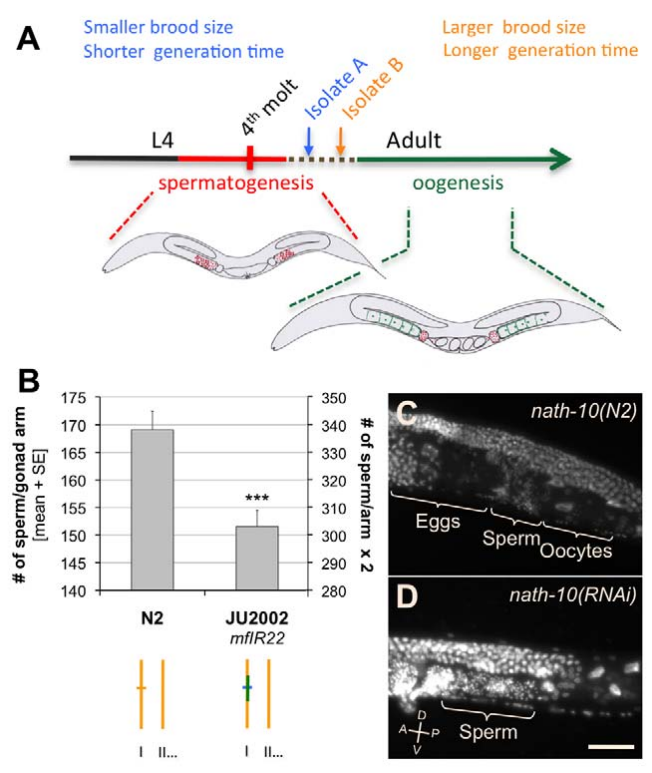
Role of *nath-10* in germ line sex determination. (A) Fitness trade-off between minimal generation time and total fertility in *C. elegans* hermaphrodites. Sperm number is a limiting factor for self-brood size. A variation of self-brood size observed among wild isolates could be explained by a variation in the timing of the sperm/oocyte switch (dotted brown line). The optimal duration of spermatogenesis that maximizes the fitness likely depends on the environmental and genetic context [52],[72]. (B) Effect of the *nath-10* polymorphism on the number of sperm produced per gonad arm at 20°C (*n* = 39). One gonad arm per animal was scored. We found no significant difference in the number of sperm produced by the anterior and posterior arms (Mann-Whitney-Wilcoxon test, *p* = 0.5). The number per gonad arm was thus doubled on the *y*-axis on the right for direct comparison with brood size (Figure 6B). The phenotypes of the two strains were compared using Mann-Whitney-Wilcoxon test: *** *p*<0.001. (C–D) Gonad arms of adult hermaphrodites fixed 1 d after sexual maturity and stained with DAPI. (C) In wild-type adults, maturing oocytes are located distally to the spermatheca and present a characteristic size and shape. Spermatozoa are located mainly in the spermatheca, but some may be driven into the uterus by newly fertilized eggs. Hyper-condensed sperm nuclei can be easily distinguished using DAPI staining. The uterus contains developing embryos. (D) *nath(RNAi)* leads to complete sterility and a partially penetrant absence of differentiated oocytes. Sperm-like cells spread throughout the proximal arm of the gonad. The animal also displays a Protruding Vulva phenotype. Bar: 40 µm.

A simple hypothesis to explain *nath-10* effects on lifetime fecundity and age at maturity was that both resulted from the regulation of spermatogenesis duration in young hermaphrodite adults (Figure 7A). In *C. elegans* hermaphrodites, spermatogenesis occurs between the late L4 and early adult stage and is followed by an irreversible switch to oogenesis. The number of sperm approximates the total number of progeny in the absence of mating with males, and is a limiting factor for lifetime fecundity. Therefore, a longer spermatogenesis leads both to an increased number of self-progeny and to a delayed oogenesis onset, creating a trade-off between brood size and age at maturity [51],[52]. In order to test whether the *nath-10* polymorphism affected the timing of the sperm-oocyte switch, we compared the number of sperm produced in N2 to that of an introgressed *nath-10(haw6805)* line. N2 did generate about 10% more sperm than the JU2002 introgressed line (Figure 7B), consistent with the observed difference in brood size and an effect on sperm-oocyte switch (Figure 6B). In addition, *nath-10(RNAi)* applied to adult hermaphrodites led to complete sterility of all progeny and to an absence of oocytes at high penetrance (Figure S4A–D), confirming a role of *nath-10* in germ line development. In these animals, sperm cells were often spread throughout the proximal gonad as visualized by DAPI staining, probably because of the absence of oocytes normally pushing them into the spermatheca (Figure 7C–D, Figure S4C–D). We thus conclude that the mutation to the *nath-10(N2)* allele resulted in an increase in sperm number in the laboratory N2 strain, which displays a larger brood size than most wild isolates [53].

### Selective Advantage of the *nath-10(N2)* Allele in Competition Assays

The above results strongly suggested that the *nath-10* polymorphism could affect fitness. The fact that the N2 allele appeared in the laboratory gave us the unique opportunity to test whether it could contribute to the adaptation of the N2 strain to its environment. Indeed, the specific environment where this genetic variation arose is better known, controlled, and reproducible than natural habitats. Therefore, we were able to perform competition experiments between strains carrying different alleles of *nath-10* in culture conditions resembling those when the derived *nath-10(N2)* allele first appeared.

**Figure 8:**
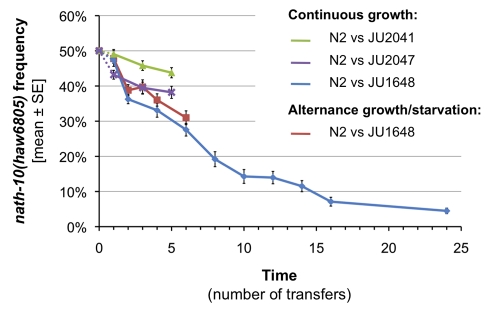
Competitive advantage of N2 over an introgressed line carrying the *nath-10(haw6805)* allele. N2 was competed in alternance of growth and starvation against JU1648 (red curve) and in continuous growth conditions against JU1648 (blue curve), JU2041 (green curve), and JU2047 (purple curve) strains. Cultures were transferred every ≈42 h for continuous growth and every week for the alternance with starvation. All experiments were started with equal numbers of synchronized individuals from each strain in 40 replicates. The frequency of *nath-10* alleles was assessed at different time points by quantitative pyrosequencing. In all cases, the frequency of the *nath-10(haw6805)* allele significantly decreased through time (comparisons between transfer 1 and 5 with Mann-Whitney-Wilcoxon rank sum test, *p* = 0.02249 for N2 versus JU2047 and *p* = 0.01797 for N2 versus JU2041, see Table S7 for statistical analysis of the N2 versus JU1648 experiments). The competitions of N2 against JU2041 or JU2047 were performed at the same time, but not in parallel to the N2 versus JU1648 experiments. The genotype of JU2041 and JU2047 in the vicinity of *nath-10* is shown in Figure S5A.

N2 was competed for several generations against JU1648 (*mfIR16[nath-10(haw6805)]*) on 6 cm diameter culture plates in two different growth conditions, namely continuous growth or alternation of growth and starvation (40 replicates per treatment). Starting at 50%, the frequency of *nath-10(haw6805)* individuals decreased across generations in both culture conditions (Figure 8). Indeed, after 24 transfers to new culture plates in continuous growth conditions (≈12 generations), JU1648 frequency reached 4.5%. The allele dynamics over generations fitted a 25.9%±4.5% selective advantage of the N2 strain. In the starvation treatment, its selective advantage was estimated to be 13.5%±8.7%. Note that these values are estimates based on approximating the number of generations per transfer. The number of generations and the treatment both presented a significant effect on the frequency of JU1648 individuals in a generalized linear model, while the interaction term was not significant (Table S7).

The competitive advantage of N2 could either be due to the *nath-10(N2)* allele or to another polymorphism in the introgressed region of JU1648. To distinguish between these two possibilities, N2 was competed in continuous growth conditions against two other strains (JU2041 and JU2047) with very fine introgressions of *nath-10(haw6805)* into the N2 genetic background. *nath-10(haw6805)* is the only allele shared by JU2041, JU2047, and JU1648 that differs with N2 (Figure S5A). Furthermore, JU2041 is expected to present only two nucleotide polymorphisms with N2, namely the *nath-10* allele and an intergenic SNP that is specific to LSJ1 (Table S2 and Figure S5A). The frequencies of JU1648, JU2041, or JU2047 individuals all decreased when competed against N2 (Figure 8), which strongly suggests that *nath-10(N2)* contributes to the increased fitness of N2. In the experiments with JU2041 and JU2047, the selective advantage of N2 per generation was respectively 9.8%±8.2% and 9.6%±8.3%. The difference with the 25.9% estimated from the independent experiment with JU1648 may be due to environmental factors, very recent mutations, epigenetic effects, or another polymorphism in the larger introgressed region of JU1648. A candidate is the coding polymorphism in the *gld-2* gene, which also appeared during N2 laboratory culture [38]. Indeed, while the finer introgressions in JU2041 and JU2047 carry the derived *gld-2(N2)* allele (Table S2), JU1648 kept the wild *gld-2(haw7249)* allele. *gld-2* is known to regulate germ line development [54],[55]. The presence of *gld-2(haw7249)* may also explain possible phenotypic differences between JU1648 and other introgressed lines (not significant in our analyses, but reproducibly observed), such as a stronger reduction of fertility in JU1648 (Figure 6B).

To conclude, these experiments show that the derived *nath-10(N2)* allele confers a strong competitive advantage in two different laboratory conditions. This result raises the possibility that *nath-10(N2)* evolved as an adaptation to the laboratory environment, although it is impossible to show this with certitude.

## Discussion

Using a quantitative genetic approach, we identified a polymorphism in the *nath-10* gene involved in the cryptic evolution of *C. elegans* vulval cell fate specification, as well as another candidate polymorphism in the *npr-1* gene. Both polymorphisms affect traits related to fitness in addition to their cryptic role in vulval development. We showed that a coding change in the essential gene *nath-10* confers a strong competitive advantage in the laboratory environment and may have evolved as an adaptation of the *C. elegans* reference strain N2 to its laboratory environment. This polymorphism subtly affects *nath-10* activity, leading to quantitative regulation of sperm number and egg laying rate in adult hermaphrodites. Therefore, we propose that cryptic variation does not necessarily accumulate neutrally, but may evolve through selection acting on its pleiotropic effects.

### Genetic Architecture of Cryptic Variation in Robust Systems

Phenotypic evolution is characterized in many phylogenetic lineages by periods of stasis followed with rapid diversification that do not correlate with genetic divergence. This non-linear relationship between genetic and phenotypic diversity is best explained by the expression of standing cryptic variation when a strong environmental or genetic perturbation occurs. The release of cryptic genetic variation in stressful conditions could facilitate adaptation to novel environments, as suggested by a recent study in which an artificial ribozyme population with accumulated cryptic variation displayed faster adaptation to a new substrate than a population without standing cryptic genetic variation [56]. Importantly, in this experiment, the increased evolutionary rate relied on extensive epistasis among cryptic polymorphisms. While individual mutations do not strongly affect fitness, rare beneficial combinations of several alleles occurred at higher frequency in the population that previously accumulated cryptic genetic variation [56]. Studying the genetic architecture of cryptic variation (i.e., its prevalence, the direction of its effects, and the epistasis between loci) and its molecular nature in more natural systems is thus crucial to assess its potential evolutionary role.

Cryptic genetic variation has previously been mapped for flowering traits in wild teosintes [17] and for the determination of the somatic sex in two *C. elegans* strains [7]. Both studies showed a polygenic basis for cryptic variation of these developmental traits, with alleles of opposite effects found in the same wild strain and no significant epistatic interactions between loci. These results confirmed experimentally that cryptic variation was prevalent in robust systems. However, the underlying molecular polymorphisms were not identified. In a higher resolution but less global study, association mapping was used to identify at the nucleotide level some of the polymorphisms responsible for cryptic variation of photoreceptor differentiation in wild *Drosophila melanogaster* lines [18]. A significant part of the phenotypic variation revealed with a dominant gain-of-function *Egfr* allele was explained by several interacting SNPs located at the *Egfr* locus itself. Interestingly, the rarer alleles tended to display more severe effects, suggesting that cryptic variation may be affected by purifying selection.

More recently, several QTLs for which the effect on growth rate depended on both activity of the Hsp90 chaperone and culture conditions were detected in a cross between a laboratory strain and a wild isolate of *Saccharomyces cerevisiae*. Four causative polymorphisms were subsequently mapped to the gene level [19]. A major difference with our study is that yeast growth rate is much less robust to environmental and genetic variation than the *C. elegans* vulval index. The *C. elegans nath-10* polymorphism most probably arose in conditions where it did not affect the vulva, whereas it is not clear whether the QTLs detected in yeast accumulated cryptically regarding growth rate.

In our study, we detected few QTLs, whose effect on vulval induction depended both on the presence of a sensitizing mutation and on temperature. Other loci of smaller effect were probably missed due to low power of our analysis, as suggested by the fraction of genetic variance that still remains unexplained. However, we were able to precisely map one major effect QTL to a coding polymorphism in the *nath-10* gene and another to the region of *npr-1*. The two QTLs display opposite additive effects on vulval induction, with *nath-10(haw6805)* increasing vulval index and *npr-1(g320)* (or another closely linked allele) decreasing it. The role of these genes in vulval induction was previously unknown, showing that cryptic variation does not necessarily affect the same genes that were found to affect the trait in classical genetic screens.

### Role of *nath-10* in Vulval Induction and Germ Line Development

The non-synonymous *nath-10* polymorphism affects both vulval induction and germ line development. In order to understand how vulval and germ line development are affected by this polymorphism, it will be crucial to determine the expression pattern of *nath-10*, as well as its site of action, the molecular activity of the NATH-10 protein and its binding partners.

Based on sequence similarity with orthologs, NATH-10 belongs to the GNAT superfamily of protein N-acetyltransferases. The best conserved part between human NAT10 and *C. elegans* NATH-10 corresponds to the putative N-acetyltransferase domain, making it likely that NATH-10 possesses a protein acetyltransferase activity. The amino-acid change caused by the *nath-10* polymorphism is located in this domain. Histones are well-known acetyltransferase substrates and their acetylation is usually associated with transcriptional activation [57]. NATH-10 could thus regulate gene expression through histone acetylation. However, other proteins can be acetylated and constitute potential targets for NATH-10. For instance, the activity of signaling pathways can be controlled by acetylation of some of their components, as is the case for Wnt signaling [58]. Vulval induction can be modulated through a change in the activity of the EGF/Ras, Delta/Notch, or Wnt/βcat signaling pathways in Pn.p cells [20]. We did not detect a significant effect of the *nath-10* polymorphism on expression of the *egl-17::cfp* reporter of the EGF/Ras pathway activity (unpublished data), either because the *nath-10* polymorphism acts at other levels in the signaling network or because of too little power to detect small reporter variations.

NATH-10 could affect vulval induction and germ line development either cell-autonomously or not. A transcriptional *nath-10::gfp* reporter [59] is expressed in many tissues during larval stages or adulthood, including the Pn.p cells and intestine. Germ line expression could not be detected, yet this may be due to germ line transgene silencing. It is possible that NATH-10 functions in a single tissue to control both vulval and germline development. For instance, four proteins required in the germ line for spermatogenesis (FBF-1, FBF-2, FOG-1, and FOG-2) were shown to inhibit vulval induction through translational repression of the *egf/lin-3* mRNA in the germ line [60]. Further work will be required to study the biological functions of the essential *nath-10* gene and to understand how these functions are affected by the *nath-10* coding polymorphism.

The inactivation of *nath-10* either with the null allele *tm2624* or by RNAi indicates that the coding polymorphism only affects a subset of all gene functions. Indeed, *nath-10(tm2624)* leads to fully penetrant embryonic lethality at the homozygous state. Depending on the intensity of the treatment, *nath-10(RNAi)* causes complete developmental arrest at the L1 stage or strong sterility accompanied by partially penetrant absence of oogenesis and diverse gonad malformations. These defects are consistent with previous results showing that *nath-10(RNAi)* led to abnormal expression of sex-specific gonadal markers [43]. Remarkably, total inactivation of *nath-10* causes sterility, while the partial loss-of-function caused by the *nath-10(N2)* allele increases fertility.

### Evolution of the N2 Laboratory Strain of *C. elegans*

The introduction of wild isolates into the environment of the laboratory strongly impacts their evolutionary trajectory and especially may increase their rate of phenotypic evolution [61]–[66]. The laboratory is a novel and usually more uniform and benign environment, with altered dynamics of population growth compared to natural habitats. Some adaptations to laboratory conditions were phenotypically characterized in *Drosophila* species but the underlying genetic bases were not identified [67],[68]. In *C. elegans*, only recently were the consequences of the early laboratory evolution of the N2 reference strain considered [34],[49]. N2 evolved a preference for high O_2_ and low CO_2_ concentrations on food, which strongly affects the behavior of the laboratory strain compared to wild isolates. This phenotypic variation was associated with two polymorphisms in the *npr-1* and *glb-5* genes [34]. Two other laboratory strains of *C. elegans* and one strain of *C. briggsae* that were cultivated for years at high population density display parallel evolution for insensitivity to pheromone-induced dauer formation [38].

Here, we show that the laboratory-derived N2 allele of *nath-10* confers a strong selective advantage in competition assays performed in laboratory conditions. Direct assessment of the adaptive role of the *nath-10* polymorphism was possible thanks to fine introgressions of *nath-10(haw6805)* from different wild isolates into the N2 background and to direct quantification of allele frequencies by pyrosequencing. This method avoids the use of a tester fluorescent strain whose fitness might be affected by the presence of the reporter gene [69],[70]. Despite its strong positive fitness effects in laboratory conditions, the *nath-10(N2)* allele would likely be outcompeted in the long term in wild conditions, since it modifies an otherwise broadly conserved amino-acid.

Different traits may explain the fitness effect of the *nath-10* polymorphism in the laboratory environment. First, the *nath-10* polymorphism finely modulates the number of sperm produced in young adult hermaphrodites, with the N2 allele resulting in a 10% increase. As expected, this effect on sperm production is associated with a longer minimal generation time and an increased lifetime fecundity in N2, two phenotypes with opposite effects on population growth rate, resulting in a fitness trade-off [51],[71]. Using a different assay to evaluate fitness (the “eating race”), a 50% increase in sperm production was previously shown to be a disadvantage in the N2 genetic background [51]. Our results, which are not inconsistent with this observation, suggest that a 10% decrease of brood size relative to N2 is also a disadvantage. Therefore, the total number of sperm produced by N2 hermaphrodites seems to have evolved toward a new optimum in the laboratory. The optimal sperm number is likely to depend on environmental conditions and on the genetic background [52],[72]. Indeed, theoretical modeling and experiments based on competition between genotypes obtained through artificial mutagenesis indicated that production of more sperm was favored in some environmental conditions but not others [72]. Importantly, hermaphrodite fertility is not always sperm-limited: sperm are produced in excess when worms are grown on compost or other food than *E. coli* [73] and temperature variations may reduce brood size independently of sperm number [72]. Therefore, the production of more sperm should be selectively favored in laboratory strains maintained on *E. coli* at 20°C, compared to wild isolates for which sperm number may not be limiting fertility. Consistently, most wild *C. elegans* isolates display a smaller brood size than the N2 strains from the *Caenorhabditis* Genetics Center [53],[74]. In addition, the earlier frozen “ancestral N2” strain presents a lower brood size than the standard N2 strain (D. Gems, personal communication). Both N2 strains carry the *nath-10(N2)* allele, suggesting that sperm number may have repeatedly increased in the laboratory through the fixation of several successive mutations.

Although this increased sperm production could be involved in the adaptation of N2 to the laboratory environment, it is probably not the only factor contributing to the competitive advantage conferred by the *nath-10(N2)* allele. Indeed, a 10% increased sperm number that presents both positive and negative effects on fitness can hardly explain a 10% to 25% selection coefficient. Moreover, for an increased lifetime fecundity to be advantageous, the adults must be able to produce all their progeny before being eliminated, which was rarely the case in our competition assays due to the frequency of transfers.

A second character that may contribute to the strongly adaptive role of the *nath-10* polymorphism in laboratory conditions is the rate of egg laying. Indeed, *nath-10(N2)* can confer up to 20% faster egg laying in the middle of adult reproductive life, which is expected to directly increase fitness. Since *nath-10(RNAi)* leads to strong oogenesis defects, the effect of the *nath-10* polymorphism on egg laying rate must also be mediated through a subtle regulation of *nath-10* activity in oogenesis. Finally, uncharacterized phenotypes might also participate in the competitive advantage of *nath-10(N2)* in the laboratory environment.

### Role of Pleiotropy in the Evolution of Cryptic Genetic Variation

Our results suggest that pleiotropic selection may play an important role in the evolution of cryptic genetic variation. Pleiotropic selection should occur when several characters share common genetic regulators and the evolution of one of these characters is driven by selection acting on another character [75],[76]. In the present case, the *nath-10* polymorphism that cryptically affects vulval development may have evolved due to pleiotropic selection on sperm production and egg laying rate.

Our results open interesting avenues for thinking about the relationship between pleiotropy and the evolution of robust systems. Robustness to mutations could contribute to alleviate the so-called “cost of complexity” according to which complexity decreases the rate of adaptation due to pleiotropy [77]. Indeed, the more genetic robustness is widespread in biological systems, the less deleterious are the pleiotropic effects of a random mutation, on average. Conversely, non-cryptic pleiotropic effects influence the accumulation of genetic variation. Cryptic mutations with pleiotropic effects should on average be more readily eliminated by natural selection than strictly cryptic (neutral) polymorphisms, while at the same time those whose non-cryptic effects are positively selected should accumulate at a faster rate than strictly cryptic polymorphisms. Thus, pleiotropy should in theory restrain and bias the accessible genotypic space for cryptic genetic variation. The prevalence and the evolutionary impact of cryptic genetic variation could thus be influenced by the distribution of pleiotropy [78].

Finally, a central assumption of evo-devo is that adaptive mutations mainly affect cis-regulatory regions because of a more restricted pleiotropy compared to coding changes [79],[80]. This idea is currently debated due to the accumulation of empirical data showing that phenotypic evolution involves both regulatory and coding mutations [81],[82]. In this context, the *nath-10* polymorphism is remarkable as an example of adaptive evolution that affects a coding sequence and at the same time displays restricted pleiotropy. Indeed, this polymorphism only affects a subset of all traits altered by the inactivation of the essential gene *nath-10*. The restricted pleiotropy of a coding mutation can be explained either by an altered interaction of the protein with tissue-specific factors or by different degrees of genetic robustness among the phenotypes regulated by the protein. The latter case applies concerning vulval cell fate pattern versus the timing of sperm-oocyte transition. In the future, molecular identification of cryptic variation in other systems will be required to determine its degree of pleiotropy and its cis-regulatory versus coding nature.

## Materials and Methods

### Strain Genotypes and Maintenance for Phenotypic Analysis

Recombinant strain genotypes are listed in Tables S1 and S2. Transgenic strains are listed in the section on transgenesis below. Strains were thawed less than six generations before phenotypic observation to avoid mutation accumulation. The strains were bleached at the first generation after thawing to eliminate possible contaminants. Well-fed strains were maintained at 20°C on standard 55 mm NGM plates, all poured the same day and seeded with the same liquid culture of *Escherichia coli* OP50 for food. Adults were shifted on fresh plates at the relevant temperature one generation prior to observation. Strains were scored on the same day in parallel for each experiment or replicate.

### Vulval Index

The number of Pn.p cells that adopted a vulval fate was scored in L4 larvae, as described previously [83]. Briefly, worms were mounted on pads of 5% noble agar, 10 mM sodium azide in M9. The number and orientation of cells of the vulval epithelium were observed under Nomarski optics (100× objective). These cell lineage outputs at the L4 stage allowed us to infer the number of Pn.p cells that were induced at the L3 stage. For a given strain, the vulval index represents the mean number of induced cells in different animals. The wild-type vulval index is 3, and upon perturbation can range from 0 to 6 (when P3.p to P8.p cells are all induced).

### QTL Analyses

The *let-23(sy1)* allele was previously introgressed into the genetic background of several wild isolates [10]. JU605 and JU606 carry this artificial mutation in the N2 and AB1 backgrounds, respectively. Sixty recombinant inbred lines (RILs) were generated from a cross between JU605 hermaphrodites and JU606 males. F1 cross-progeny were distinguished from F1 self-progeny by genotyping two RFLP markers (*pkP1071* and *pkP5082*) in the corresponding F2 larvae. The RILs were initiated from 60 F2 individuals randomly isolated among the offspring of 3 F1 cross-progeny. The 60 lines were isogenized through 12 generations of selfing of one random animal per generation and frozen down.

RILs were genotyped for 50 single nucleotide polymorphisms (SNPs) distinguishing N2 and AB1 and distributed along most of the genome (Table S1). Most SNP markers were chosen from genotyping data on *C. elegans* wild isolates [49], except *mfP11*, *mfP12*, and *mfP13*, which were deduced from the whole-genome sequencing of AB1 (L. Stein et al., personal communication). Forty-eight SNP markers were genotyped by the Integragen company using SNPlex technology and two SNP markers were genotyped by pyrosequencing using a PyroMark Q96 ID instrument (Biotage). All genotypes were determined from purified genomic DNA prepared using the DNeasy Blood & Tissue Kit (Qiagen).

The vulval index was determined in the 60 RILs grown at 20°C (two replicates) and at 25.5°C (two replicates). For each replicate, five gravid adults were picked on 3 plates per RIL and all lines were cultured in parallel at the appropriate temperature (20°C or 25.5°C). After 52 (20°C) or 40 h (25.5°C), all plates were transferred to 7°C in order to stop further development of the progeny past the L4 stage. Scoring at the L4 stage could be performed up to 2 d after transfer at 7°C. Two experimenters scored the vulval index of 30 L4 larvae per RIL for the 60 lines, following the method described above. This treatment allowed us to score all RILs in parallel and thus minimize environmental effects.

From these genotypic and phenotypic data (Table S1), QTL analyses were performed using composite interval mapping [84] in QTL cartographer v1.16 [85],[86], with model 4 for markers used as cofactors. Under this model, the most significant marker on each chromosome (except for the tested chromosome) is used to control for genetic background. Four analyses were performed using each phenotypic replicate (two at 20°C and two at 25.5°C). A fifth analysis was performed using as trait value the difference in vulval index between 25.5°C and 20°C (in the replicates that were scored in parallel) to map the phenotypic plasticity to temperature change.

### Construction of Near-Isogenic Lines (NILs)

Several near-isogenic lines (NILs) were established to finely map the chromosome I QTL. Several hermaphrodites from the three RILs that presented the highest vulval index at 20°C (RIL5, RIL15, and RIL38; Table S2) were crossed to JU605 males. In F2, L4 larvae that displayed a normal vulval invagination under the dissecting microscope were backcrossed to JU605 males. Three rounds of vulval phenotype selection and backcross in F2 were followed by 13 generations of selfing to obtain homozygous near-isogenic lines. Worms were grown at 20°C instead of 25.5°C during NIL construction because the temperature effect had not yet been found. The NILs that showed the highest vulval index at 25.5°C shared a region of AB1 genotype in the center of chromosome I (Table S2).

To further narrow down the QTL region, we selected 20 lines with a recombination event in the QTL interval on chromosome I. Hermaphrodites from NIL11 (cross A) and NIL13 (cross B) were crossed to JU605 males. 312 (cross A) and 320 (cross B) F2 progeny were isolated and selfed until the F5 generation. One F5 adult from each plate was genotyped for two SNP markers surrounding the QTL region: markers *haw6686* and *haw7143* for cross A and markers *mfP11* and *haw7143* for cross B (AB1 alleles in NIL11 and NIL13). Twenty lines (10 for each cross) presented the N2 allele at one marker and the AB1 allele at the other marker. The 20 lines were genotyped for several additional SNPs in the QTL region and scored for vulval index at 25.5°C (Table S3). This restricted the QTL to a 186 kb interval. Three such lines were used for further studies (JU1620 and JU1624 from cross A and JU1610 from cross B). SNP genotyping in the NILs was performed by pyrosequencing using a PyroMark Q96 ID instrument.

To confirm the effect of the *nath-10* polymorphism, we established a NIL (JU2000) from a cross between LSJ1 hermaphrodites and JU605 males. *nath-10(haw6805)*; *let-23(sy1)* F2 progeny were backcrossed 10 times to JU605 males to introgress the LSJ1 *nath-10* allele into the N2 background, with molecular selection of *nath-10(haw6805)*.

To generate the JU1961, JU1648, JU2003, and JU2002 strains, the *let-23(sy1)* mutation was removed by crosses of N2 males with JU1620, JU1648, JU1610, and JU2000, respectively. The *let-60(n1046)* mutation was introduced into the JU1624 background by a cross with JU601 males to generate the JU1756 line. The construction of JU601 and JU473 strains was previously described [10]. Finally, JU2041 and JU2047 strains present finer introgressions of *nath-10(haw6805)* from the LSJ1 and AB1 backgrounds into N2. These two strains were obtained by crossing JU2002 or JU2003 hermaphrodites to N2 males and by selecting for F5 individuals that carried the *nath-10(haw6805)* allele and a N2 allele at an adjacent marker (*mfP17* for JU2041 and *haw6686* for JU2047). The *let-23(sy1)* allele was introduced into JU2041 and JU2047 backgrounds by crossing the two lines with JU605, yielding the JU2133 and JU2135 strains. From these crosses, we also selected as a control individuals presenting the *nath-10(N2)* genotype that founded the JU2134 and JU2136 strains. The genotypes of all NILs are presented in Table S2.

### N2/AB1 Polymorphisms in the Chromosome I QTL Region

N2 and AB1 sequences corresponding to the 183 kb QTL region (region spanning from 5190795 bp to 5376700 bp on chromosome I) were compared to find all polymorphisms present in this genomic interval. N2 sequence was obtained from wormbase (www.wormbase.org) and AB1 sequence was obtained through whole-genome Illumina sequencing of the AB1 strain used to build the JU606 strain (L. Stein et al., personal communication). Nine SNPs could be detected by comparing these N2 and AB1 sequences, including the *nath-10* polymorphism. Sanger sequencing revealed that six of these SNPs were false positive due to errors in the AB1 sequence (at positions 5315591, 5330853, 5333385, 5333386, 5336190, and 5340310) and two were false positive due to errors in the N2 sequence (at positions 5221052 and 5301101). In addition, the AB1 sequence presented 149 gaps in the QTL region (ranging from 1 to 50 bp) that were not covered by the Illumina sequencing. Sanger sequencing of the 149 gaps in AB1 revealed two additional SNPs in the QTL region named *haw6786* and *mfP14* (see Table S2 for alleles).

### Genotyping of the *nath-10* Polymorphism

SNP genotyping based on pyrosequencing technology (PyroMark Q96 ID instrument from Biotage) was used to select the homozygous *nath-10(haw6805)* F2 (or F3) individuals in the different lines described above and to determine the distribution of the *nath-10* polymorphism in *C. elegans* wild isolates. Single worm PCRs were performed in 50 µl reactions [87] using GoTaq DNA polymerase (Promega), with the following primers: forward 5′-GCCGGGAACGAGGAAAAGTCAAATG-3′ and biotinylated reverse 5′-[Btn]TTCGGACTCACTGTTCC-3′. The purification of single-stranded PCR amplicons and the pyrosequencing reactions were performed according to the manufacturer's instructions using the following sequencing primer: 5′-GTTCGAGTCCTTTCAG-3′.

### Overexpression of *nath-10*

Transgenic lines with non-integrated arrays were established according to standard techniques [88]. The coding sequence of *nath-10* plus upstream and downstream intergenic regions were amplified by PCR from JU605 or JU606 genomic DNA using Phusion DNA polymerase and the following primers: forward 5′-ATGGCCAATGATTGGGATGCTG-3′ and reverse 5′-CTGAAGATTACGGTACGAGGTCTCG-3′. PCR products were gel-purified using the Wizard SV Gel and PCR Clean-Up System (Promega). Three different mixes of DNA were independently injected into the gonad of adult worms: mix 1 and mix 2 contained, respectively, 0.2 ng/µl of *nath-10* PCR product from JU605 or JU606, 10 ng/µl of pWD47, and 140 ng/µl of pBluescript; mix 3 contained 10 ng/µl of pWD47 and 140 ng/µl of pBluescript and served as a negative control. pBluescript phagemid was used as carrier DNA and the *Pmyo-2::DsRed* construct (pWD47 plasmid) as coinjection marker. *nath-10* PCR products were injected at only 0.2 ng/µl because higher concentrations (≥1 ng/µl) led to sterility of the F1 progeny. Each mix was injected into JU605 and JU1620 and for each of the four possible combinations two independent transgenic lines were scored for vulval index.

Overexpression of *nath-10(N2)* in a *nath-10(N2)* endogenous context: JU1953: *nath-10(N2)I*; *let-23(sy1)II*; *mfEx54[nath-10(N2), myo-2::DsRed]*; JU1954: *nath-10(N2)I*; *let-23(sy1)II*; *mfEx55[nath-10(N2), myo-2::DsRed]*. Overexpression of *nath-10(haw6805)* in a *nath-10(N2)* endogenous context: JU1887: *nath-10(N2)I*; *let-23(sy1)II*; *mfEx46[nath-10(haw6805), myo-2::DsRed]*; JU1888: *nath-10(N2)I*; *let-23(sy1)II*; *mfEx48[nath-10(haw6805), myo-2::DsRed]*. Overexpression of *nath-10(N2)* in a *nath-10(haw6805)* endogenous context: JU1951: *nath-10(haw6805)I*; *let-23(sy1)II*; *mfEx52[nath-10(N2) myo-2::DsRed]*; JU1952: *nath-10(haw6805)I*; *let-23(sy1)II*; *mfEx53[nath-10(N2) myo-2::DsRed]*. Overexpression of *nath-10(haw6805)* in a *nath-10(haw6805)* endogenous context: JU1889: *nath-10(haw6805)I*; *let-23(sy1)II*; *mfEx47[nath-10(haw6805), myo-2::DsRed]*; JU1890: *nath-10(haw6805)I*; *let-23(sy1)II*; *mfEx49[nath-10(haw6805), myo-2::DsRed]*.

For each line, five culture plates containing 10 adult worms expressing *myo-2::DsRed* were grown at 25.5°C for 44 h. The vulval index was scored on progeny that express *myo-2::DsRed*, and also on progeny that spontaneously lost the transgene and did not express *myo-2::DsRed* (for lines injected with mix 1 and 2). This additional control allowed us to distinguish zygotic effects from maternally or epigenetically inherited effects by directly comparing the vulval index of animals sharing the same mother.

### RNAi against *nath-10*

The animals were fed with bacteria from the Ahringer library [89] expressing dsRNA targeted against *nath-10* or with control bacteria containing the empty RNAi vector L4440. In the case of JU605, JU606, and JU1624, five adults were placed on four RNAi plates for each worm line and for each bacterial strain in two replicate experiments. After 44 h at 25.5°C, L4 progeny were scored for vulval induction. RNAi against *nath-10* could not be applied for several generations because it led to strong sterility of the progeny.

In the case of N2, several adults were grown at 25°C on RNAi plates in parallel to control plates. The worms had to be grown at 25°C so that the progeny reach the adult stage. Indeed, when applied to adult worms cultured at 20°C, *nath-10(RNAi)* led to complete arrest of the progeny at the L1 stage. After 100 h of growth at 25°C, adult progeny were either directly mounted on pads of 5% noble agar, 10 mM sodium azide in M9 (Figure S4), or fixed and mounted in DAPI Vectashield (Figure 7C and 7D). Images of the gonads were acquired on a Zeiss AxioImager M1 microscope equipped with a Photometrics CoolSnap ES CCD camera driven by the Metaview 6.3r7 software.

### Interaction between Different Alleles of *nath-10*

Vulval induction of heterozygous F1 cross-progeny (*nath-10(N2)/nath-10(haw6805)*) was compared to that of control homozygous cross-progeny to determine the dominance relationship between the two alleles. Practically, we scored reciprocal crosses between JU605 and JU606 or JU1620 as well as control crosses of JU605, JU606, and JU1620 strains. For each cross, one 8-d-old (sperm depleted) hermaphrodite was mated to five young males on each of 10 plates. The mating plates were grown at 25.5°C for 40 h and the vulval induction index of L4 hermaphrodite progeny was scored from plates that showed about 50% of males.

The effect of the null allele *nath-10(tm2624)* on vulval induction was assessed in heterozygous *nath-10(N2)/nath-10(tm2624)* animals carrying the *egfr/let-23(sy1)* sensitizing mutation. First, as the *tm2624* deletion is embryonic lethal, it was maintained in an heterozygous state using the *szT1* balancing translocation between chromosomes I and X [90]. The JU1982 strain (*tm2624/szT1[lon-2(e678)] I*; *+/szT1 X*) was obtained by crossing heterozygous *nath-10(N2)/nath-10(tm2624)* hermaphrodites generated by the National BioResource Project to AF1 males (*+/szT1[lon-2(e678)] I; dpy-8(e1321) unc-3(e151)/szT1 X*). JU1988 (*tm2624/szT1[lon-2(e678)] I; let-23(sy1)/let-23(sy1) II; +/szT1 X*) was then established from a cross between JU1982 and JU605. The JU1989 strain (*+/szT1[lon-2(e678)] I; let-23(sy1)/let-23(sy1) II; dpy-8(e1321) unc-3(e151)/szT1 X*) obtained by crossing JU605 to AF1 was used to control for the effect of the *szT1* translocation on vulval induction. The *tm2624* allele leads to a deletion of 618 bp (replaced by a TCA triplet) in the *nath-10* coding region that could be followed by PCR using the forward 5′-CGACCACCATAGCCCATTGAC-3′ and reverse 5′-GGTCGTGGACGTGGAAAGTCT-3′ primers. The vulval induction index of JU1988 and JU1989 was scored at 25.5°C as described above.

### Assessment of *nath-10* SynMuv Effect

The JU1803 line (*mfIR16[nath-10(haw6805)]; lin-15A(n767)*) was obtained from a cross between MT1806 (*nath-10(N2); lin-15A(767)*) hermaphrodites (from CGC) and JU1648 (*mfIR16[nath-10(haw6805)]*) males. F2 individuals with the correct genotype were selected after pyrosequencing of *nath-10* and Sanger sequencing of *lin-15*. The same method was employed to establish the JU1804 line (*mfIR16[nath-10(haw6805)]; lin-15B(n744)*) from a cross between MT2495 (*nath-10(N2); lin-15B(n744)*) hermaphrodites (from CGC) and JU1648 males. The MT1642 (*nath-10(N2); lin-15AB(n765)*) strain (from CGC) was used to show the effect of a double mutant of class A and B SynMuv genes. The vulval induction index was scored in strains grown in parallel at 25.5°C.

### NATH-10 Sequence Alignment

A local alignment was performed on the Expasy proteomics server using the BLOSUM62 comparison matrix with default settings (gap open penalty = 12, gap extension penalty = 4) and displayed using the LALNVIEW graphical viewer program. GNAT-related N-acetyltransferase domain (amino-acids 558–753) and a putative ATP binding domain (amino acids 284–291) were identified using bioinformatic tools [39].

### Effect of *npr*-*1* Polymorphism

The *npr-1(g320)* allele was introduced into the genetic background of JU605 (*nath-10(N2); let-23(sy1); npr-1(N2)*) and JU1624 (*nath-10(haw6805); let-23(sy1); npr-1(N2)*) strains. To this end, DA650 (*npr-1(g320)*) hermaphrodites (from CGC) were crossed to JU605 males. Several egg laying defective F2 progeny were isolated at the L4 stage and a single F3 progeny from a plate that presented a majority of egg laying defective and clumping adults was used to establish the JU1617 strain (*nath-10(N2); let-23(sy1); npr-1(N2)*). JU1617 hermaphrodites were then crossed to JU1624 males and a similar strategy was employed to generate the JU1650 line (*nath-10(haw6805); let-23(sy1); npr-1(g320)*) with pyrosequencing of the *nath-10* allele in the F3 progeny. The vulval induction index was scored in JU605, JU1617, JU1624, JU1650, and JU606 strains grown in parallel at 25.5°C.

### P12 to P11 Cell Fate Transformation

The frequency of P12 to P11 cell fate transformation was scored in strains carrying the *lin-45(n2018)/raf* mutation combined with either the N2 or the AB1 *nath-10* allele. In these strains, the reduction of Ras pathway activity can lead the P12 cell to adopt a P11-like cell fate. The penetrance of this phenotype was assessed by counting the number of P11.p-like and P12.pa-like cells at the L4 stage under Nomarski optics (100× objective) based on the morphology and position of cell nuclei [91].

### Dauer Entry at 27°C

Strains were synchronized by 2 h of egg laying on several plates at 20°C and progeny were incubated for 44 h at 27°C. On each plate, dauer and non-dauer larvae were enumerated under the dissecting microscope based on the characteristic morphology of dauers.

### Age at Reproductive Maturity

The strains were synchronized by 1 h of egg laying of 20 adults on one plate, for two consecutive generations (because the second generation appeared to lay eggs that were better synchronized). For each strain, 20 larvae were isolated on separate 55 mm culture plates 24 h after the second synchronization. From 58 h post-synchronization, plates were checked every hour for laid eggs until all isolated hermaphrodites reached reproductive maturity. The age at maturity had been calculated as the difference between the time when the first eggs were observed and the time when the parent itself was laid (estimated as the middle time point of the 1 h egg laying period). All plates were subsequently kept at 20°C for fertility assessment. The animals were grown at 20°C.

### Fertility

Brood size was assessed in three replicate experiments, following the protocol described for the scoring of the age at maturity. The three replicates were treated similarly, except that the strains were synchronized for one generation in two replicates and for two consecutive generations in the last one (both age at maturity and fertility are shown here for this last replicate). After maturation, adults were transferred twice per day on fresh plates during 3 d and then once per day until no more fertilized eggs were laid. The total brood size of a given adult was calculated as the sum of the progeny that reached the L3–L4 stage, as counted on each plate. For each strain, 20 adults were grown in parallel at 20°C and the rare ones that died prematurely were discarded from the analysis.

### Maximal Egg Laying Rate

The transfer of adults to new plates during the fertility experiments allowed us to estimate the egg laying rate at different broad periods of their reproductive life. This rate was calculated by dividing the number of progeny that reached the L3–L4 stage on a given plate by the duration of adult egg laying on that plate. The egg laying rate varied during adulthood and usually reached its maximum in the period spanning 20 to 30 h after sexual maturity.

### Sperm Count

The total number of spermatids produced per gonad arm was counted in young N2 and JU2002 adults (39 per strain) fixed in −20°C ethanol and stained with DAPI. Briefly, the strains were synchronized by 2 h of egg laying on 10 plates per strain with 20 adults per plate and the resulting progeny were fixed after 62 h of culture at 20°C. Worms were then mounted on Vectashield DAPI and Z-series fluorescence images of selected gonad arms were acquired on a Zeiss AxioImager M1 microscope (100× objective). Individual spermatid DNA could be clearly distinguished for only one gonad arm per animal (the one closest to the coverslip). In order to score gonad arms where spermatogenesis was completed, but where fertilization did not yet start, we selected animals where ovulation started in only one gonad arm and scored the other arm. After acquisition, the Z-series (20 images spaced by 1 µm) for each animal was projected onto a single plane using ImageJ 1.43r and spermatids were counted on the screen.

### Competition Assay

Four competition experiments involving different strains and culture conditions were performed to assess the adaptive value of the *nath-10(N2)* allele in the laboratory. In the first two assays, individuals of the N2 strain were allowed to compete against individuals of the JU1648 strain (Table S3) that present the *nath-10(haw6805)* allele introgressed from the AB1 background into the N2 background. In these experiments, worms were grown in parallel in two different environmental conditions that could potentially reflect the culture conditions when the *nath-10* polymorphism first appeared, namely continuous growth on OP50 or alternation of growth and starvation. In addition, two competition experiments were performed with different strains in continuous growth conditions in order to control for the effect of the introgressed regions. For this purpose, two near-isogenic lines were constructed as described above and allowed to compete against N2. JU2041 (*mfIR24(I;LSJ1>N2*) was obtained from a cross between JU2002 and N2, while JU2047 (*mfIR27(I;AB1>N2)*) was obtained from a cross between JU2003 and N2 (Table S2). *nath-10(haw6805)* is the only polymorphism with N2 shared by JU1648, JU2041, and JU2047. For each assay, 40 replicates were cultured in parallel on 6 cm diameter NGM dishes seeded with one drop of OP50. All replicate cultures were started with seven L4 individuals of each of the two competing strains placed in the center of the *E. coli* lawn.

For the continuous growth treatment, populations of worms were transferred to fresh plates by cutting out a chunk of agar at fixed intervals before bacteria depletion. A 0.5×0.5 cm^2^ piece of agar containing at least 100 individuals was cut out from the middle of the bacterial lawn of old plates and deposited at the edge of the bacterial lawn on fresh culture plates. The first transfer was made 78 h after the beginning of the experiments to allow sufficient growth of the initially small population. The next transfers were performed approximately every 42 h.

For the growth/starvation treatment, populations were transferred by cutting out an agar chunk (containing at least 200 individuals) once a week at a fixed time point. Worms were fed for the 2–3 d following each transfer and then starved for the next 4–5 d. *C. elegans* populations were grown at 20°C for all competition assays.

For each replicate, the proportion of N2 individuals was assessed at different time points during the competition experiment (Figure 8) through quantification of the frequency of *nath-10* alleles using pyrosequencing. For the population in continuous growth, allele frequencies were determined from old plates before starvation and after the transfer to the new dish. For the growth/starvation treatment, subcultures were initiated each week in parallel to the experimental populations to determine allele frequencies from non-starved plates. For DNA preparation, 5 µl of M9 suspensions containing about 100–300 mixed-stage individuals from each culture plate were mixed with 25 µl of worm lysis buffer and proteinase K (100 µg/ml). After 1.5 h of lysis at 60°C and 15 min of inactivation at 95°C, 0.5 µl of worm lysate were used as PCR template. The primers and PCR conditions were as described above for *nath-10* polymorphism genotyping. Allele frequencies were obtained from the height of the pyrogram peaks using the Allele Quantification tool supplied with the pyrosequencing software of Biotage.

To measure the accuracy of this quantification method, a standard curve was performed with different known proportions of alleles. The correlation between observed and real allele frequencies exceeded 0.993 and the average standard deviation calculated from a triplicate of observed frequencies was 4%.

### Statistical Analyses

Except for QTL analyses, all statistical tests were performed with R (http://www.r-project.org/). For the competition assays between N2 and JU1648, we used a generalized linear model (GLM) to assess the variation of *nath-10(haw6805)* allele frequency over time and across growth conditions. This response variable was assumed to follow a Gaussian distribution and a log link function was used. Effects included in the model were *generation* treated as a number, *treatment* treated as a nominal variable, and the *generation×treatment* interaction. The effect of *replicates* was nested within *treatment*. For the competition experiments involving JU2041 and JU2047 strains, allele frequencies were assessed at only a few time points. Therefore, we compared the *nath-10(haw6805)* frequency at the first and at the last time points using a Mann-Whitney-Wilcoxon test. The relative fitness of *nath-10(N2)* over *nath-10(haw6805)* was estimated for all assays using the method described previously for selection in haploid organisms, with correction for continuous growth when necessary [92]. Our populations behave as haploid as very few males are present during the competition experiments. The mean generation time in the continuous growth conditions was estimated to be 86 h, which corresponds roughly to 0.5 generations per transfer. For the alternated growth/starvation treatment, the growth rate was considered to be one generation per transfer (worms start to starve about 72 h after transfer to a new plate).

## Supporting Information

Figure S1 Temperature sensitivity and variability of the vulval phenotype of JU606. Each colored bar represents the mean vulval index of the progeny of individual hermaphrodites and the error bar represents SE among parents (*n* = 3–4 parents, 16–26 progeny per parent). Progeny that share the same grandmother are similarly colored. Letters above dashed lines indicate significance groups after pairwise Mann-Whitney-Wilcoxon comparisons with Holm-Bonferroni corrections. Treatments that do not share the same letter are statistically different (*p*<0.05). Two effects are detected. First, two sub-cultures of JU606 show significant differences in vulval index. This hereditary phenotypic modification of JU606 was observed several times and in both directions, and could be due to either genetic or epigenetic variation. Second, both JU606 cultures are affected by temperature in the same way, whereas JU605 is not significantly affected. The vulval index of JU606 increases when the growth temperature exceeds a critical threshold between 24°C and 25.5°C. The number of induced vulval cells being sensitive to unidentified factors, we systematically used a stringent strain thawing protocol prior to each scoring. Strain genotypes are schematized as in Figure 3.
(TIF)

Figure S2 Cryptic effect of an *npr-1(g320)* introgression on vulval induction. Introgression of the AB1 allele *npr-1(g320)* into the backgrounds of JU605 or JU1624 decreases the vulval index. Comparison of JU1650 and JU606 shows that the QTL detected on chromosomes I and X are not the only one involved in the cryptic genetic variation. Statistical significance using Mann-Whitney-Wilcoxon tests: * *p*<0.05, ** *p*<0.01, *** *p*<0.001 (*n* = 56–68). Strain genotypes are schematized as in Figure 3, with horizontal lines on chromosomes I, II, and X representing *nath-10* alleles, *let-23(sy1)*, and *npr-1* alleles, respectively.
(TIF)

Figure S3 Vulval induction effect of different *nath-10* allelic combinations. (A) Dominance of the *nath-10(haw6805)* allele over the *nath-10(N2)* allele for vulval induction in the presence of *let-23(sy1)*. Animals heterozygous for the N2 and AB1 *nath-10* alleles were obtained by crossing JU605 to either JU606 or JU1620 (*n* = 40–61). Regardless of the cross direction, heterozygous *nath-10(N2)/nath-10(haw6805)* F1 animals presented a significantly higher vulval index than homozygous *nath-10(N2)* F1 animals and the same index as *nath-10(haw6805)* F1 animals at 25.5°C. The significance of the difference with the control outcross of JU605 to itself is represented over each bar. The significance of other comparisons is shown above the dotted lines. Mann-Whitney-Wilcoxon tests: ns, non-significant, * *p*<0.05, ** *p*<0.01, *** *p*<0.001. Chromosome I genotypes are schematized as in Figure 3. (B) Effect of a single copy of *nath-10(N2)* on vulval induction. The deletion allele *nath-10(tm2624)*, which removes 618 bp spanning exons 4 to 7, was used to construct heterozygous strains expressing a single copy of *nath-10(N2)*. The JU1988 line is maintained in an heterozygous *nath-10(N2)/nath-10(tm2624)* state using the *szT1* balancing translocation between chromosomes I and X [90]. The JU1989 strain carries the *szT1* translocation in an homozygous *nath-10(N2)* context. Both strains carry the *egfr/let-23(sy1)* sensitizing mutation. The vulval index of JU1988 is not significantly different from JU1989 at 25.5°C. However, a significant decrease of vulval induction is observed in JU1989 compared to JU605, which could be explained either by an effect of the *szT1* translocation alone, by the *nath-10(tm2624)* deletion, or by a combination of both. The vulval index of homozygous *nath-10(tm2624)* animals cannot be scored due to the embryonic lethality of this null allele. Two experimental replicates are shown with dark gray (*n* = 26–60) and light gray bars (*n* = 82–90). Note the smaller scale of the *y*-axis than on panel (A). For each replicate, pairwise comparisons were performed using Mann-Whitney-Wilcoxon tests. Then, the *p* values obtained for each replicate were combined according to Fisher's method and the significance represented over the dotted lines as in (A). On the schematized genotypes of the strains (not to scale), each chromosome was represented with a specific motif to visualize the translocation.
(TIF)

Figure S4 Effect of RNAi against *nath-10* on germ line development of adult hermaphrodites, observed using Nomarski optics. (A) Proximal part of the posterior gonad arm of a wild-type animal showing an oocyte, the spermatheca filled with sperm, and embryos in the uterus. (B–D) *nath-10(RNAi)* adult hermaphrodites with complete loss of oogenesis. (B) Thin proximal gonad, characteristic of a *nath-10(RNAi)* animal without oocytes. The proximal gonad presents a granular aspect and is filled with small cells of undetermined sexual fate. (C) The proximal part of the gonad (bottom left) contains cells with a sperm-like morphology, while the sexual fate of more distal germ cells (bottom right) is unclear. (D) The gonad is filled with sperm-like cells. Residual bodies of spermatogenesis are still present in this 4-d-old adult, whereas they are normally only observed at L4 and early adult stages. Thus, spermatogenesis may persist during adulthood in *nath-10(RNAi)* animals. Alternatively, spermatogenesis might be blocked before spermatid separation or the residual bodies may not be resorbed. Letters indicate orientation as follows: A, Anterior; P, Posterior; D, Dorsal; V, Ventral. Bars: 20 µm.
(TIF)

Figure S5 Genotypes of the NILs used in the competition assays and vulval induction index of related lines sensitized with the *let-23(sy1)* mutation. (A) Genotypes of strains used for competition assays and scoring of vulval induction index. JU2041 present the *nath-10(haw6805)* allele introgressed from LSJ1 into the N2 background. JU2047 and JU1648 carry two independent introgressions of the *nath-10(haw6805)* allele from AB1 into N2. *nath-10(haw6805)* is the only allele shared by JU2041, JU2047, and JU1648 that is different from N2. The introgressed regions of JU2133, JU2135, and JU1624 strains are, respectively, the same as JU2041, JU2047, and JU1648, but in a *let-23(sy1)* context. Finally, JU2134 and JU2136 were derived from the same crosses that yielded JU2133 and JU2135, but they are homozygous for the *nath-10(N2)* allele. They were compared to JU2133 and JU2135 as additional controls. Note that *nath-10(haw6805)* and *mfP19* are the two only genetic differences between N2 and JU2041 (apart from undetected de novo mutations). (B) Vulval induction index of the most refined NILs grown at 25.5°C. The vulval index of strains homozygous for *nath-10(N2)* are not significantly different from JU605, whereas strains homozygous for *nath-10(haw6805)* show a higher induction index whatever the original genetic background of this allele (*n* = 40). The statistical significance of the comparison of each strain with JU605 is represented above the corresponding bar (Mann-Whitney-Wilcoxon rank sum test: ns, non-significant, * *p*<0.05, ** *p*<0.01, *** *p*<0.001).
(TIF)

Table S1 Genotype and vulval phenotype of the 60 Recombinant Inbred Lines (RILs) and 28 Near-Isogenic Lines (NILs). All strains carry the *let-23(sy1)* mutation.
(XLS)

Table S2 Chromosome I genotype and vulval phenotype of the 20 lines that present a recombination in the QTL region and of the lines where the LSJ1 allele of *nath-10* was introgressed into the N2 background.
(XLS)

Table S3 Cell fate patterns of P3.p to P8.p cells in JU605, JU606, JU1620, and JU1624 strains grown at 25.5°C. The main difference among strains is not the penetrance, but the expressivity of induction defects (*n* = 30 for each strain). Letters indicate the orientation of the division of the Pn.pxx cells as follows: T, transverse (left-right); L, longitudinal (anteroposterior); U, undivided; S, fusion with the hypodermal syncytium cell hyp7, which is a non-vulval fate; ?, missing cell. Cells that are attached to the cuticle are underlined. The fate of Pn.p cells were scored from the number and position of Pn.pxx(x) nuclei. The 1° fate is colored in blue, 2° fate in red, 3° fate in yellow, 1°/2° intermediate fate in purple, 2°/3° in orange, and the fused non-vulval fate in gray. Wild-type patterns are highlighted with bold letters.
(XLS)

Table S4 The *nath-10(haw6805)* allele does not display a classic SynMuv phenotype. *nath-10(haw6805)* was crossed with either a SynMuv A (*lin-15A(n767)*) or a SynMuv B (*lin-15B(n744)*) mutation. No extra vulval cell induction was observed in either case. Therefore, *nath-10* does not seem to act as a classic SynMuv gene.
(TIF)

Table S5 Allelic distribution of *nath-10* in *C. elegans* wild strains and other species. The N2 allele (T) is only found in Bristol-derived strains, whereas the AB1 allele (C) is conserved in all other strains and species. N2 (CGC) is the CGC reference strain. N2 (ancestral) comes from a stock frozen in the Brenner lab in 1968 that was sub-cultured for about six generations before being frozen at the CGC. CB4555 and TR389 strains are likely N2 lab contaminants [34]. LSJ1 was derived from the same Bristol wild isolate than N2 and was grown for several years in axenic liquid culture in Berkeley. ^1^SNP allele determined with pyrosequencing-based genotyping. ^2^SNP allele determined from whole-genome sequencing data.
(TIF)

Table S6 Phenotypic differences between N2 and AB1 that do not involve the *nath-10* polymorphism. In N2, entry into the alternative dauer stage can be induced at low penetrance by growth at 27°C [50]. We observed that the dauer larvae frequency at 27°C is much higher in AB1 compared to N2. However, introduction of *nath-10(haw6805)* into the N2 background (JU1648 and JU2002) does not affect the penetrance of dauer entry at 27°C. The penetrance of P12 to P11 cell fate transformation in *lin-45(n2018)* mutants was previously shown to vary with the wild genetic background [10]. Consistent with this, we observed that the frequency of larvae with two P11.p-like cells is lower in JU646 (*lin-45(n2018)* in N2) compared to JU891 (*lin-45(n2018)* in AB1) at 20°C. This effect is not observed at 25.5°C nor in the JU1754 strain that carries *nath-10(haw6805)* (derived from JU1648) and *lin-45(n2018)* in the N2 background. Mann-Whitney-Wilcoxon tests were performed to compare the penetrance in N2 (or JU646) to the penetrance in the other strains: ns, non-significant; * *p*<0.05, ** *p*<0.01, *** *p*<0.001.
(TIF)

Table S7 Summary of a generalized linear model of the influence of *generation*, *treatment*, and the interaction between both on the frequency of the *nath-10(haw6805)* allele in competition assays between N2 and JU1648.
(TIF)

## Abbreviations

GLM: generalized linear model
GxE: genotype-by-environment
GxG: genotype-by-genotype
NILs: near-isogenic lines
QTL: quantitative trait locus
RILs: Recombinant Inbred Lines
