# Supplementary figures and images for: Role of Pleiotropy in the Evolution of a Cryptic Developmental Variation in Caenorhabditis elegans
Source: PLoS Biol. 2012 Jan 3;10(1):e1001230. doi: 10.1371/journal.pbio.1001230 (PMC3250502; doi:10.1371/journal.pbio.1001230)

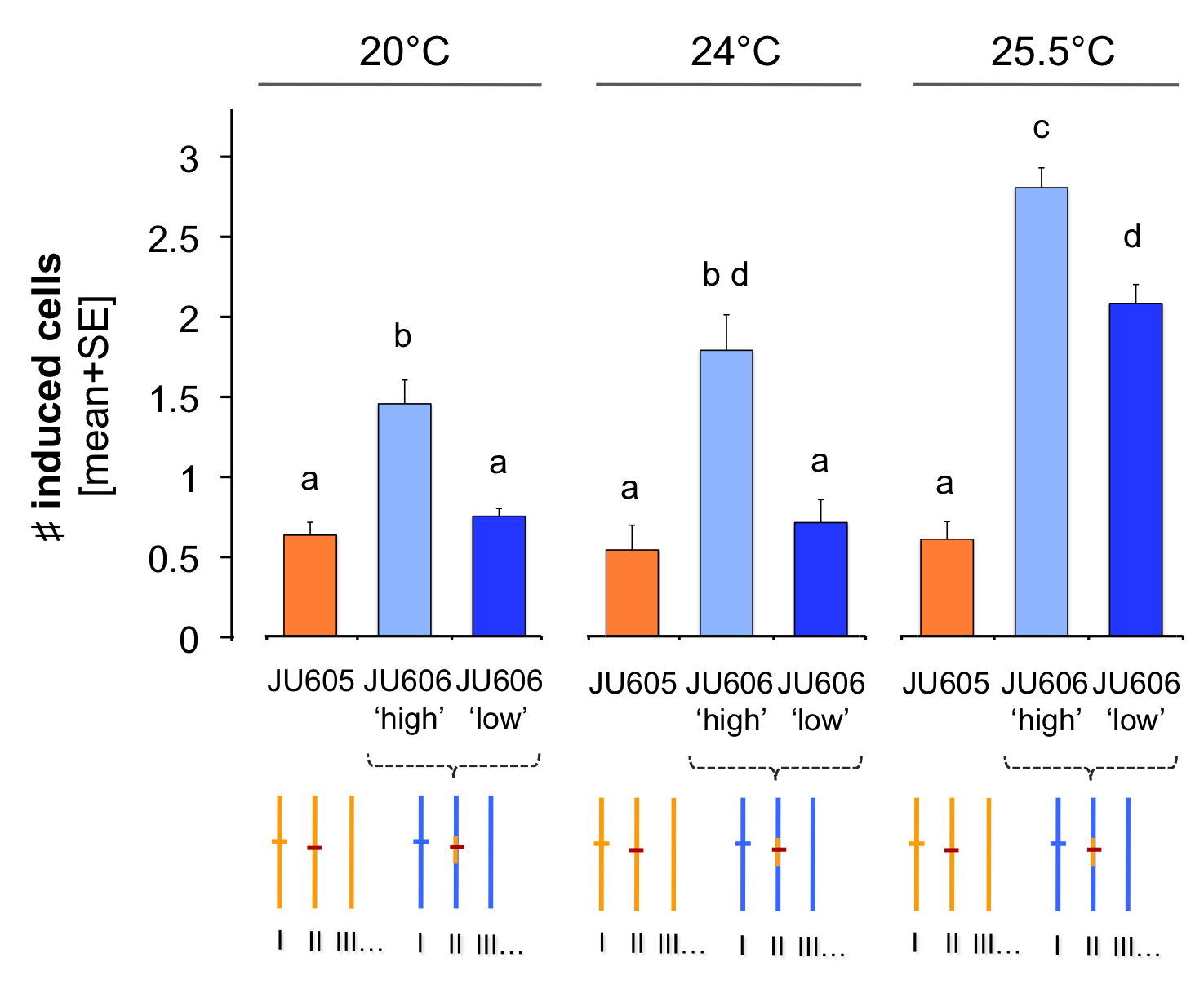

Supplement: Figure S1 — Temperature sensitivity and variability of the vulval phenotype of JU606. Each colored bar represents the mean vulval index of the progeny of individual hermaphrodites and the error bar represents SE among parents (n = 3–4 parents, 16–26 progeny per parent). Progeny that share the same grandmother are similarly colored. Letters above dashed lines indicate significance groups after pairwise Mann-Whitney-Wilcoxon comparisons with Holm-Bonferroni corrections. Treatments that do not share the same letter are statistically different (p<0.05). Two effects are detected. First, two sub-cultures of JU606 show significant differences in vulval index. This hereditary phenotypic modification of JU606 was observed several times and in both directions, and could be due to either genetic or epigenetic variation. Second, both JU606 cultures are affected by temperature in the same way, whereas JU605 is not significantly affected. The vulval index of JU606 increases when the growth temperature exceeds a critical threshold between 24°C and 25.5°C. The number of induced vulval cells being sensitive to unidentified factors, we systematically used a stringent strain thawing protocol prior to each scoring. Strain genotypes are schematized as in Figure 3. (TIF) [file pbio.1001230.s001.tif]

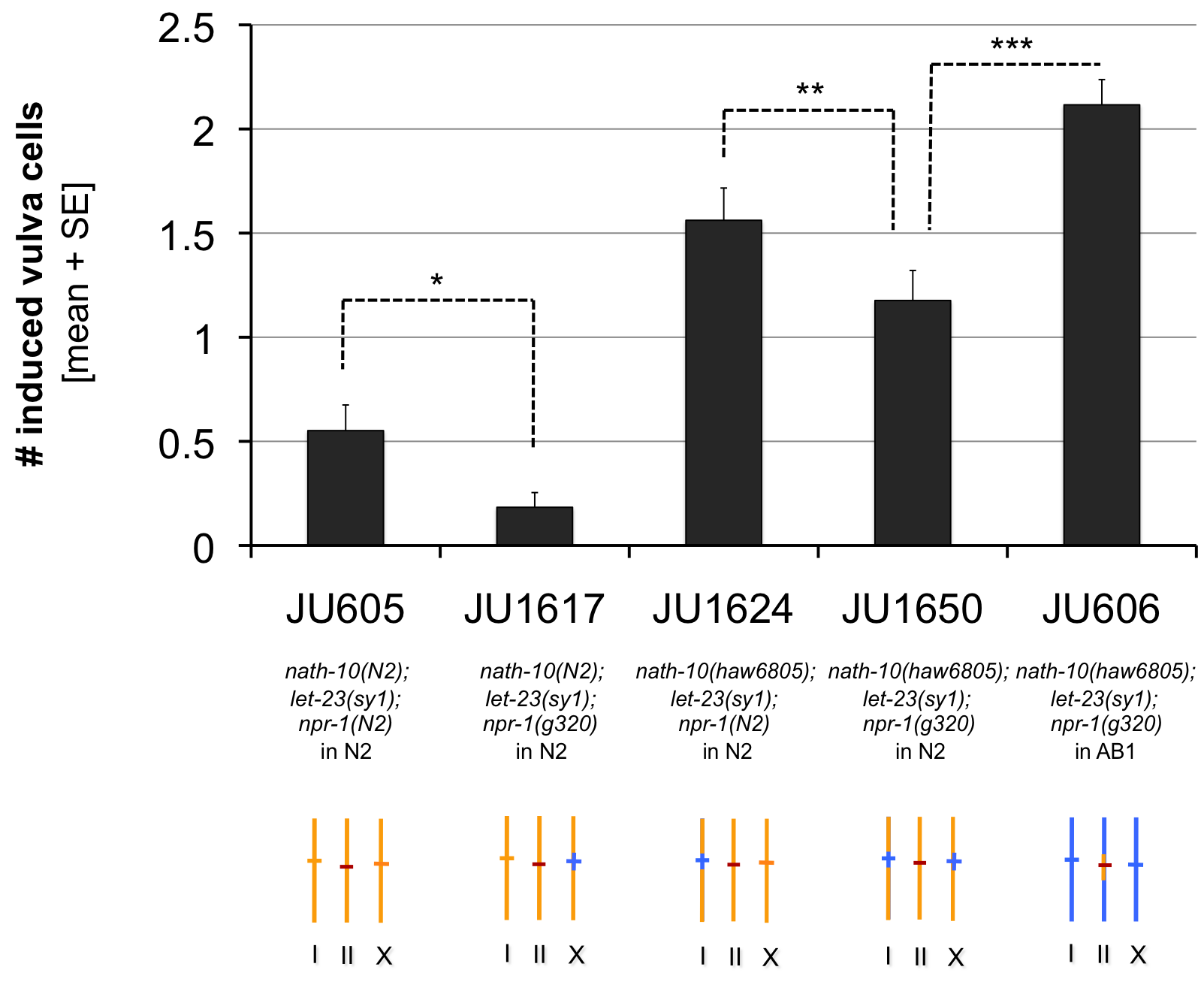

Supplement: Figure S2 — Cryptic effect of an npr-1(g320) introgression on vulval induction. Introgression of the AB1 allele npr-1(g320) into the backgrounds of JU605 or JU1624 decreases the vulval index. Comparison of JU1650 and JU606 shows that the QTL detected on chromosomes I and X are not the only one involved in the cryptic genetic variation. Statistical significance using Mann-Whitney-Wilcoxon tests: * p<0.05, ** p<0.01, *** p<0.001 (n = 56–68). Strain genotypes are schematized as in Figure 3, with horizontal lines on chromosomes I, II, and X representing nath-10 alleles, let-23(sy1), and npr-1 alleles, respectively. (TIF) [file pbio.1001230.s002.tif]

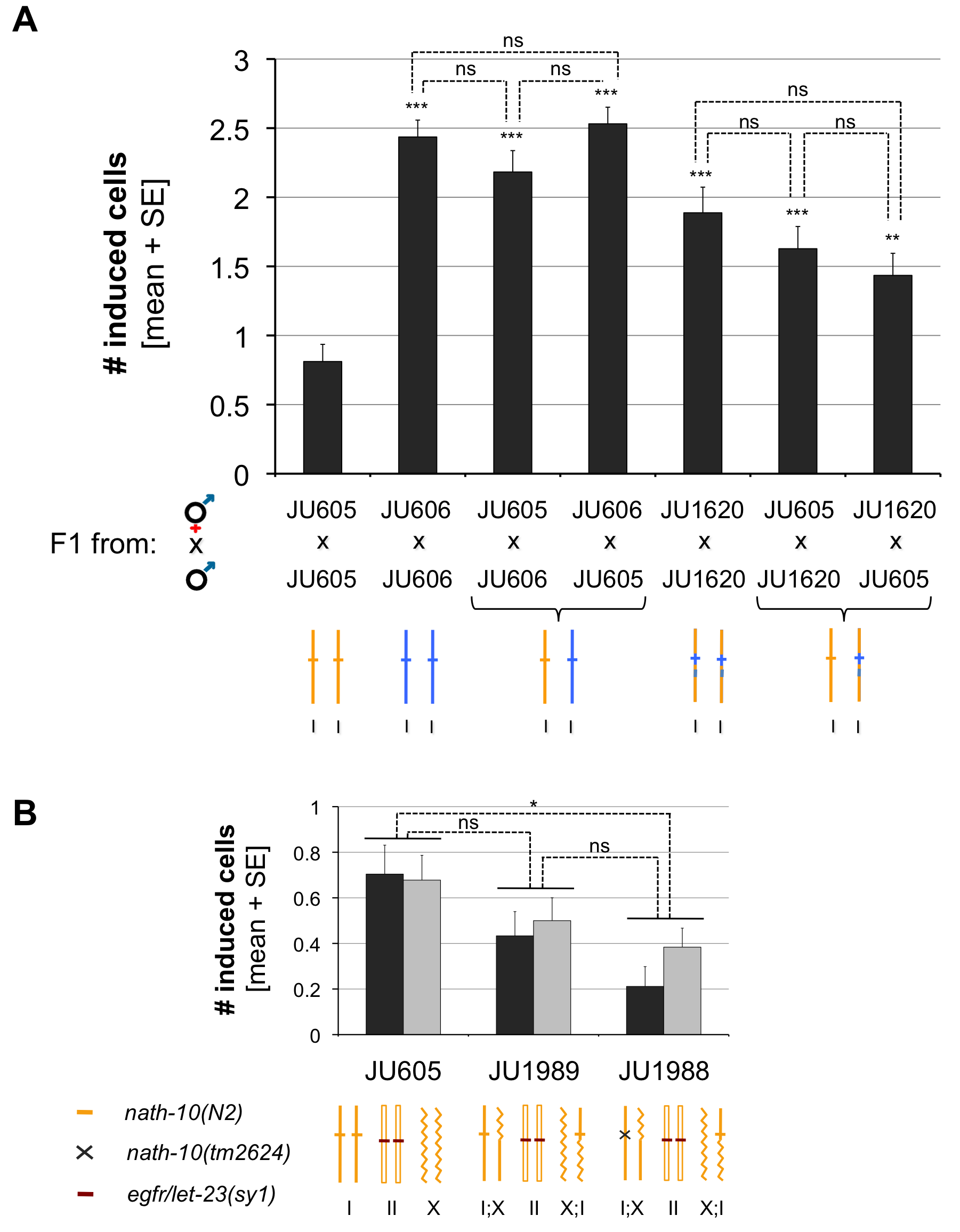

Supplement: Figure S3 — Vulval induction effect of different nath-10 allelic combinations. (A) Dominance of the nath-10(haw6805) allele over the nath-10(N2) allele for vulval induction in the presence of let-23(sy1). Animals heterozygous for the N2 and AB1 nath-10 alleles were obtained by crossing JU605 to either JU606 or JU1620 (n = 40–61). Regardless of the cross direction, heterozygous nath-10(N2)/nath-10(haw6805) F1 animals presented a significantly higher vulval index than homozygous nath-10(N2) F1 animals and the same index as nath-10(haw6805) F1 animals at 25.5°C. The significance of the difference with the control outcross of JU605 to itself is represented over each bar. The significance of other comparisons is shown above the dotted lines. Mann-Whitney-Wilcoxon tests: ns, non-significant, * p<0.05, ** p<0.01, *** p<0.001. Chromosome I genotypes are schematized as in Figure 3. (B) Effect of a single copy of nath-10(N2) on vulval induction. The deletion allele nath-10(tm2624), which removes 618 bp spanning exons 4 to 7, was used to construct heterozygous strains expressing a single copy of nath-10(N2). The JU1988 line is maintained in an heterozygous nath-10(N2)/nath-10(tm2624) state using the szT1 balancing translocation between chromosomes I and X [90]. The JU1989 strain carries the szT1 translocation in an homozygous nath-10(N2) context. Both strains carry the egfr/let-23(sy1) sensitizing mutation. The vulval index of JU1988 is not significantly different from JU1989 at 25.5°C. However, a significant decrease of vulval induction is observed in JU1989 compared to JU605, which could be explained either by an effect of the szT1 translocation alone, by the nath-10(tm2624) deletion, or by a combination of both. The vulval index of homozygous nath-10(tm2624) animals cannot be scored due to the embryonic lethality of this null allele. Two experimental replicates are shown with dark gray (n = 26–60) and light gray bars (n = 82–90). Note the smaller scale of the y-axis than on panel (A). [file pbio.1001230.s003.tif]

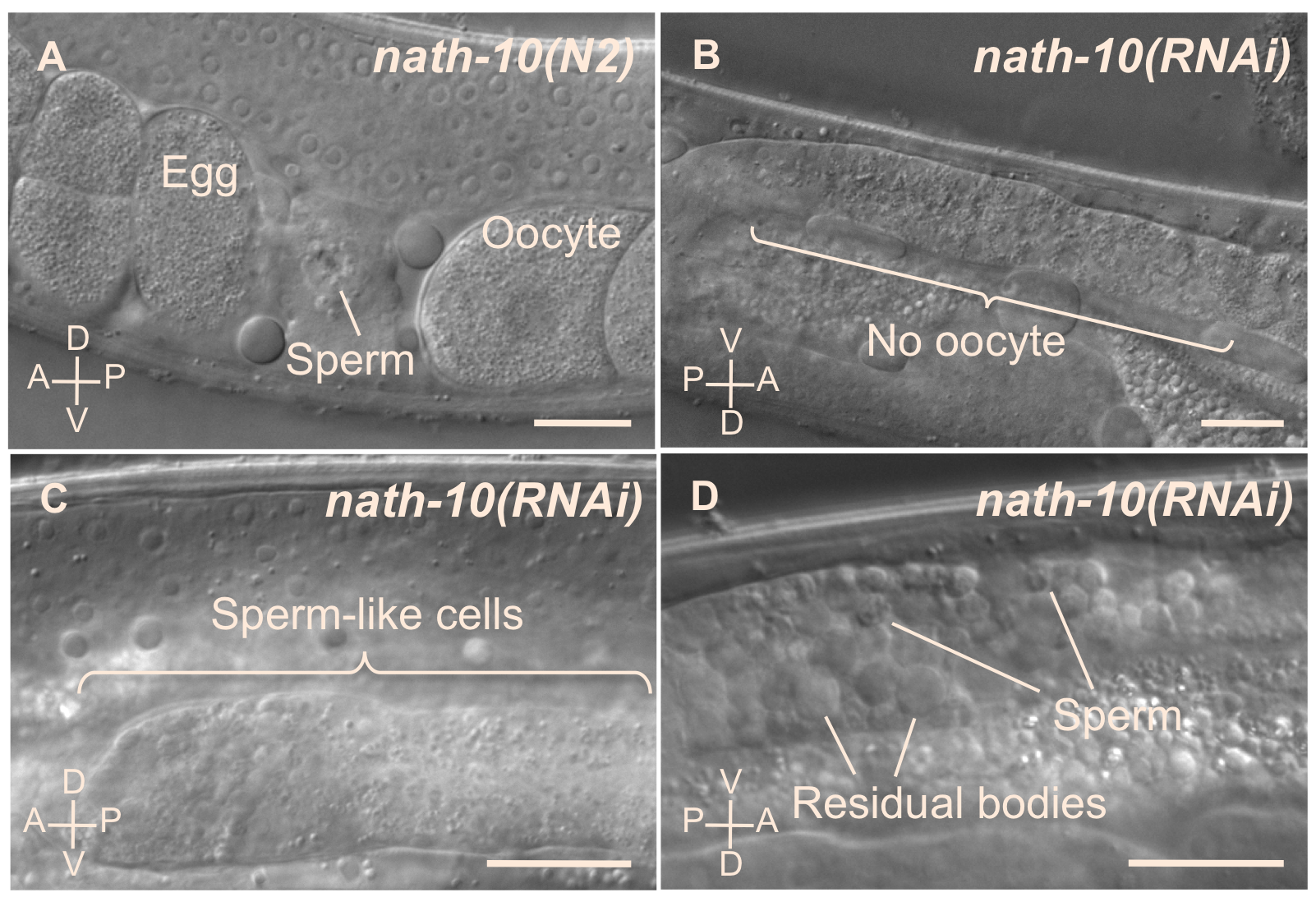

Supplement: Figure S4 — Effect of RNAi against nath-10 on germ line development of adult hermaphrodites, observed using Nomarski optics. (A) Proximal part of the posterior gonad arm of a wild-type animal showing an oocyte, the spermatheca filled with sperm, and embryos in the uterus. (B–D) nath-10(RNAi) adult hermaphrodites with complete loss of oogenesis. (B) Thin proximal gonad, characteristic of a nath-10(RNAi) animal without oocytes. The proximal gonad presents a granular aspect and is filled with small cells of undetermined sexual fate. (C) The proximal part of the gonad (bottom left) contains cells with a sperm-like morphology, while the sexual fate of more distal germ cells (bottom right) is unclear. (D) The gonad is filled with sperm-like cells. Residual bodies of spermatogenesis are still present in this 4-d-old adult, whereas they are normally only observed at L4 and early adult stages. Thus, spermatogenesis may persist during adulthood in nath-10(RNAi) animals. Alternatively, spermatogenesis might be blocked before spermatid separation or the residual bodies may not be resorbed. Letters indicate orientation as follows: A, Anterior; P, Posterior; D, Dorsal; V, Ventral. Bars: 20 µm. (TIF) [file pbio.1001230.s004.tif]

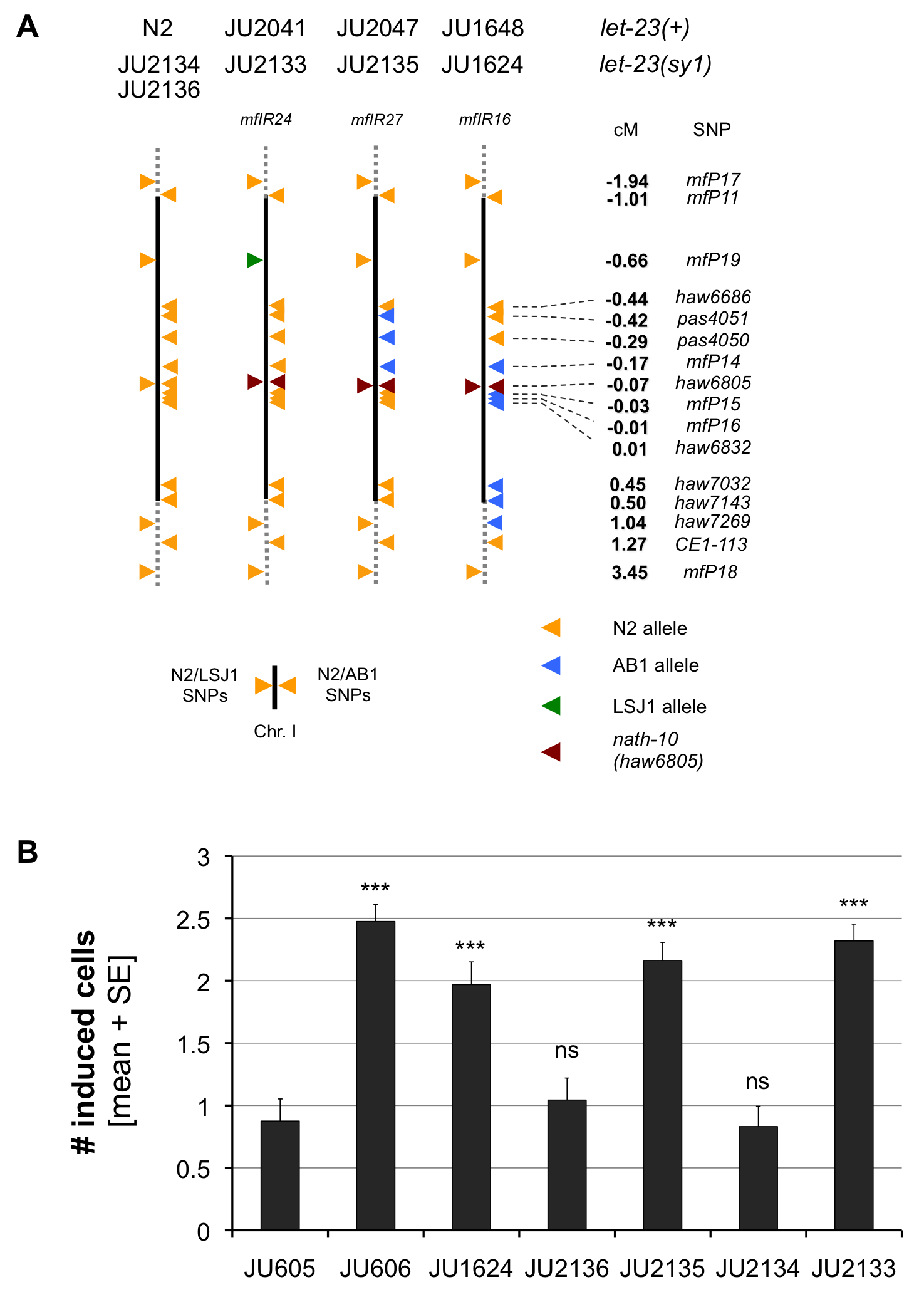

Supplement: Figure S5 — Genotypes of the NILs used in the competition assays and vulval induction index of related lines sensitized with the let-23(sy1) mutation. (A) Genotypes of strains used for competition assays and scoring of vulval induction index. JU2041 present the nath-10(haw6805) allele introgressed from LSJ1 into the N2 background. JU2047 and JU1648 carry two independent introgressions of the nath-10(haw6805) allele from AB1 into N2. nath-10(haw6805) is the only allele shared by JU2041, JU2047, and JU1648 that is different from N2. The introgressed regions of JU2133, JU2135, and JU1624 strains are, respectively, the same as JU2041, JU2047, and JU1648, but in a let-23(sy1) context. Finally, JU2134 and JU2136 were derived from the same crosses that yielded JU2133 and JU2135, but they are homozygous for the nath-10(N2) allele. They were compared to JU2133 and JU2135 as additional controls. Note that nath-10(haw6805) and mfP19 are the two only genetic differences between N2 and JU2041 (apart from undetected de novo mutations). (B) Vulval induction index of the most refined NILs grown at 25.5°C. The vulval index of strains homozygous for nath-10(N2) are not significantly different from JU605, whereas strains homozygous for nath-10(haw6805) show a higher induction index whatever the original genetic background of this allele (n = 40). The statistical significance of the comparison of each strain with JU605 is represented above the corresponding bar (Mann-Whitney-Wilcoxon rank sum test: ns, non-significant, * p<0.05, ** p<0.01, *** p<0.001). (TIF) [file pbio.1001230.s005.tif]

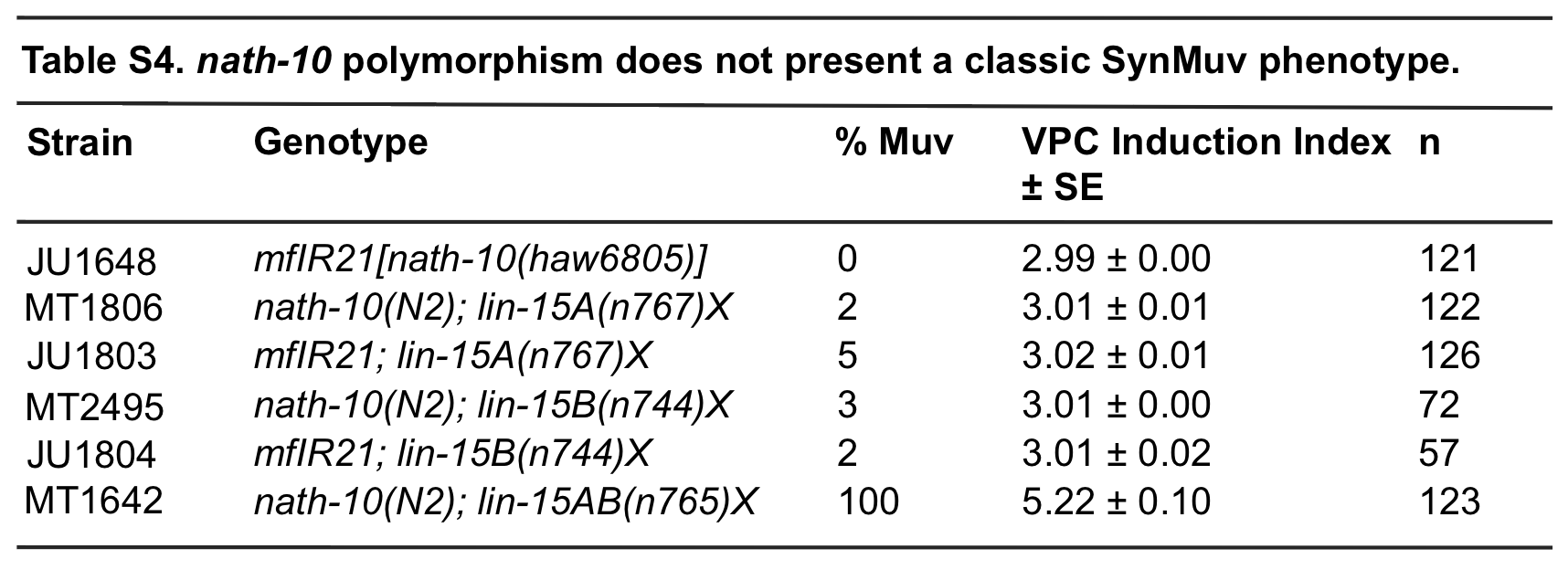

Supplement: Table S4 — The nath-10(haw6805) allele does not display a classic SynMuv phenotype. nath-10(haw6805) was crossed with either a SynMuv A (lin-15A(n767)) or a SynMuv B (lin-15B(n744)) mutation. No extra vulval cell induction was observed in either case. Therefore, nath-10 does not seem to act as a classic SynMuv gene. (TIF) [file pbio.1001230.s009.tif]

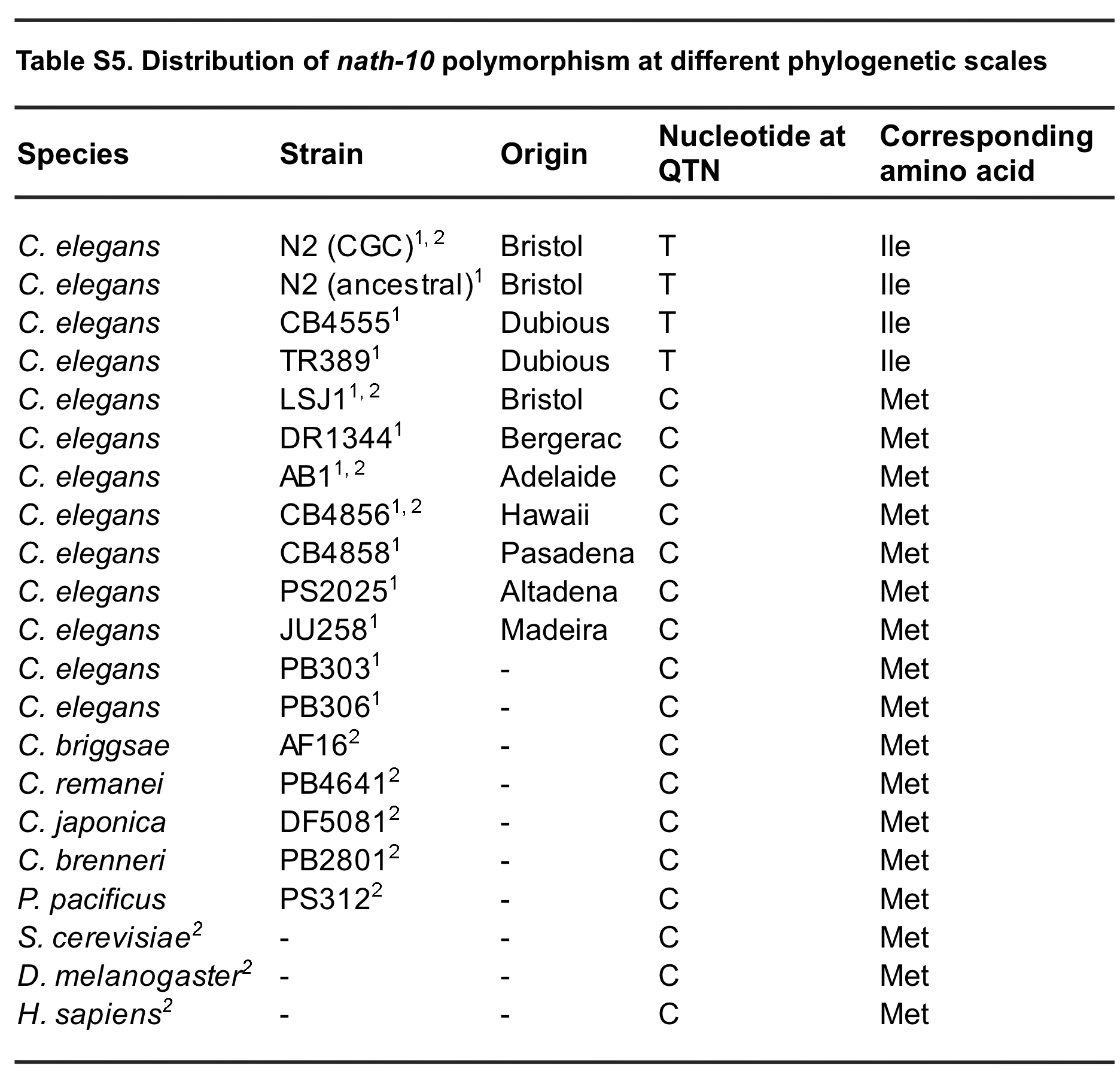

Supplement: Table S5 — Allelic distribution of nath-10 in C. elegans wild strains and other species. The N2 allele (T) is only found in Bristol-derived strains, whereas the AB1 allele (C) is conserved in all other strains and species. N2 (CGC) is the CGC reference strain. N2 (ancestral) comes from a stock frozen in the Brenner lab in 1968 that was sub-cultured for about six generations before being frozen at the CGC. CB4555 and TR389 strains are likely N2 lab contaminants [34]. LSJ1 was derived from the same Bristol wild isolate than N2 and was grown for several years in axenic liquid culture in Berkeley. 1SNP allele determined with pyrosequencing-based genotyping. 2SNP allele determined from whole-genome sequencing data. (TIF) [file pbio.1001230.s010.tif]

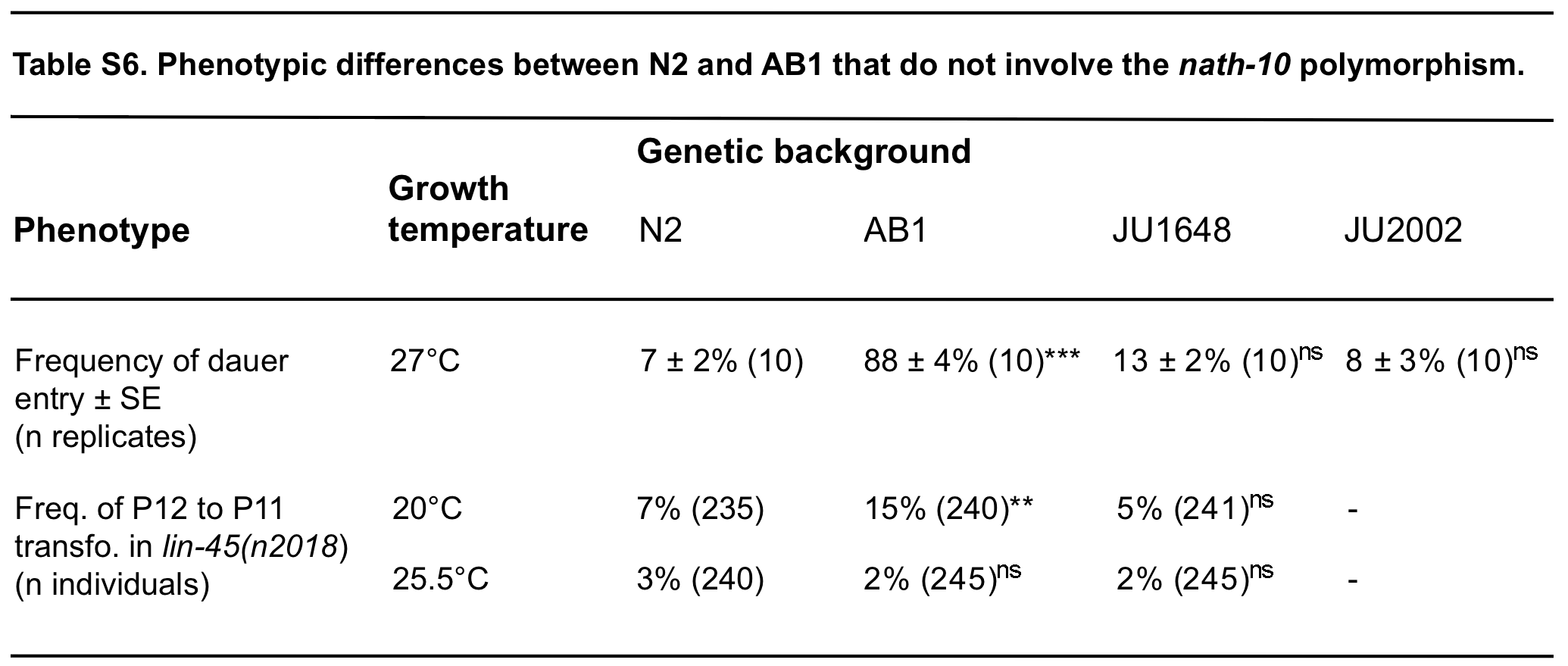

Supplement: Table S6 — Phenotypic differences between N2 and AB1 that do not involve the nath-10 polymorphism. In N2, entry into the alternative dauer stage can be induced at low penetrance by growth at 27°C [50]. We observed that the dauer larvae frequency at 27°C is much higher in AB1 compared to N2. However, introduction of nath-10(haw6805) into the N2 background (JU1648 and JU2002) does not affect the penetrance of dauer entry at 27°C. The penetrance of P12 to P11 cell fate transformation in lin-45(n2018) mutants was previously shown to vary with the wild genetic background [10]. Consistent with this, we observed that the frequency of larvae with two P11.p-like cells is lower in JU646 (lin-45(n2018) in N2) compared to JU891 (lin-45(n2018) in AB1) at 20°C. This effect is not observed at 25.5°C nor in the JU1754 strain that carries nath-10(haw6805) (derived from JU1648) and lin-45(n2018) in the N2 background. Mann-Whitney-Wilcoxon tests were performed to compare the penetrance in N2 (or JU646) to the penetrance in the other strains: ns, non-significant; * p<0.05, ** p<0.01, *** p<0.001. (TIF) [file pbio.1001230.s011.tif]

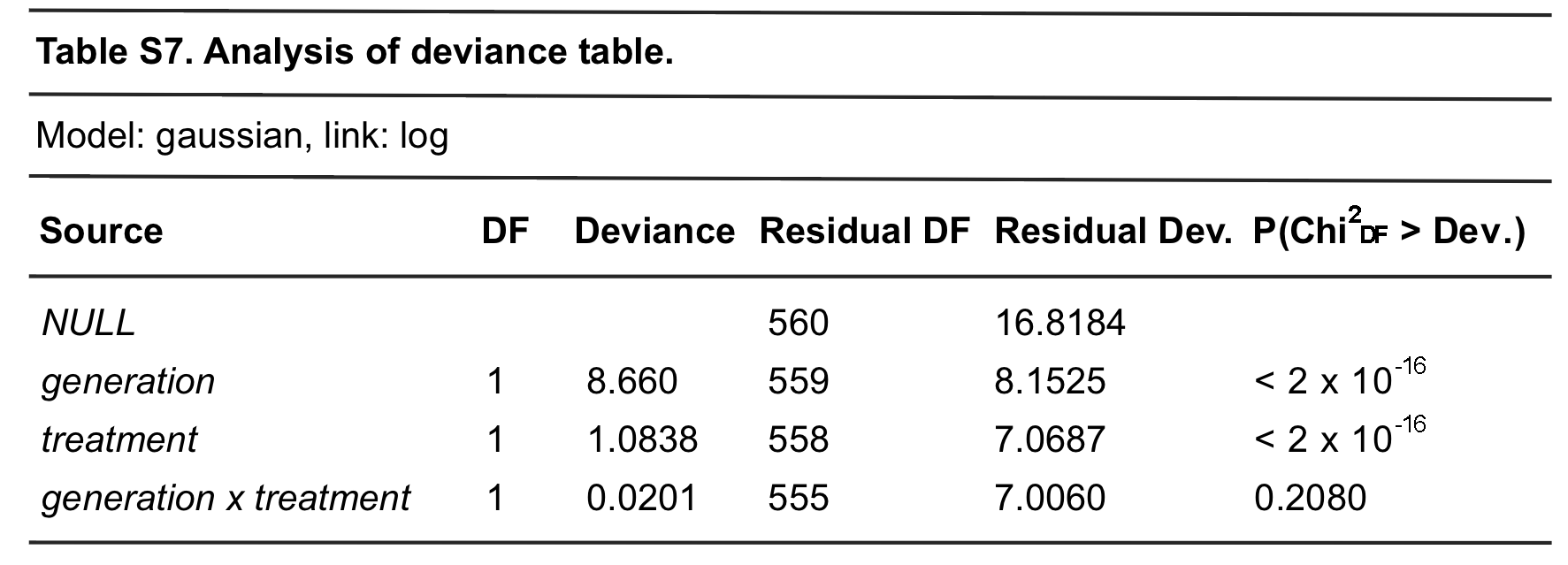

Supplement: Table S7 — Summary of a generalized linear model of the influence of generation, treatment, and the interaction between both on the frequency of the nath-10(haw6805) allele in competition assays between N2 and JU1648. (TIF) [file pbio.1001230.s012.tif]
